# Chemically induced deceleration of nuclear spin relaxation (CIDER) preserves hyperpolarization

**DOI:** 10.1126/sciadv.adx2316

**Published:** 2025-09-12

**Authors:** Josh P. Peters, Charbel Assaf, Arne Brahms, Kolja Them, Mirco Gerdsen, Rainer Herges, Jan-Bernd Hövener, Andrey N. Pravdivtsev

**Affiliations:** ^1^ Section Biomedical Imaging, Molecular Imaging North Competence Center (MOIN CC), Department of Radiology and Neuroradiology, University Hospital Schleswig-Holstein, Kiel University, Am Botanischen Garten 14, 24118, Kiel, Germany.; ^2^ Otto Diels Institute for Organic Chemistry, Kiel University, Otto-Hahn Platz 4, 24098 Kiel, Germany.

## Abstract

Gadolinium-based contrast agents revolutionized magnetic resonance imaging (MRI) by accelerating spin relaxation. In contrast, agents that decelerate relaxation were hitherto unknown. Such agents are highly desirable for metabolic imaging with hyperpolarized tracers such as ^15^N-pyridine, 1,4-^13^C_2_-succinate, and 1-^15^N-nicotinamide, where valuable polarization decays rapidly, especially at low fields during transfer between polarizer and scanner. Here, we report on a previously unrecognized effect in which the tracers’ longitudinal and transverse relaxation rates in aqueous solution are substantially reduced by adding nicotinamide, urea, glycerol, or dendrons. The impact on longitudinal relaxation is particularly pronounced at low magnetic fields and near the tracer’s p*K*_a_ where *T*_1_ can be tripled. This mitigates polarization loss during transfer, so hitherto unsuitable, fast-relaxing molecules can be used now. This way, we achieved the ^15^N hyperpolarization of nearly 30% for 1-^15^N-nicotinamide. This chemically induced deceleration of nuclear spin relaxation (CIDER) was confirmed using magnetic field–cycling experiments and offers broad potential for hyperpolarized magnetic resonance and beyond.

## Teaser

Biocompatible additives reduce nuclear relaxation and enable record-high hyperpolarization of MRI contrast agents.

## INTRODUCTION

Hyperpolarized magnetic resonance (HMR) represents a major advance in magnetic resonance imaging (MRI) by substantially enhancing sensitivity and enabling real-time metabolic imaging in humans (*1*, *2*). This technology has enabled remarkable applications, e.g., allowing the early detection of cancer before tumor formation is evident or allowing for the assessment of treatment response within just a few days of therapy initiation (*3*). This way, HMR addresses the greatest impediment of MRI, the low sensitivity, and one of the greatest needs of modern diagnostics and precision medicine, the visualization of molecular processes noninvasively before pathologies manifest.

Although dissolution dynamic nuclear polarization (dDNP) is the primary technique for human HMR, supported by numerous preclinical studies, and has immediate implications for clinical studies (*1*–*4*), parahydrogen-based technologies are also rapidly developing (*5*–*8*). Arguably, the most notable hurdle of HMR is the comparably short lifetime of signal enhancement. The lifetime (*T*_1_) of commonly hyperpolarized tracers such as ^13^C-labeled pyruvate, fumarate, urea, and acetate is about 1 min in vivo (*9*) and longer in vitro (*10*). HMR’s challenge lies in finding tracers whose relaxation is slow enough compared to the processes they probe. This constraint prohibits straightforward imaging of many interesting metabolic processes, such as glycolysis, where the metabolism is fast, but the lifetime of ^13^C-glucose-*d*_6_ is short (*T*_1_ ≈ 10 s) (*11*, *12*). Conversely, *T*_1_ for amino acids can be long, but metabolic rates are slow (*13*).

**Fig. 1.**
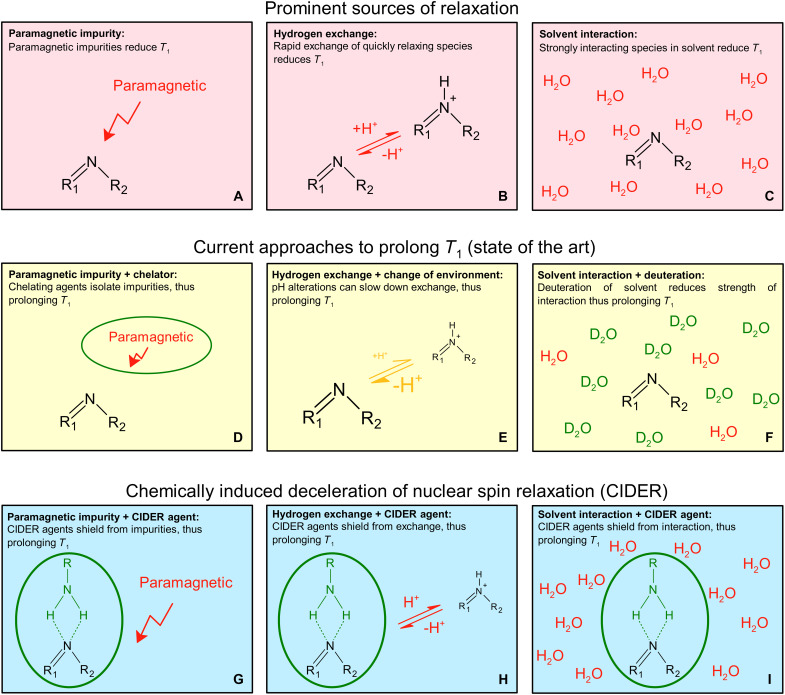
Illustration of molecular interactions affecting nuclear spin relaxation. Schematic overview of prominent sources of relaxation in liquid-state magnetic resonance [(**A**) to (**C**)], current approaches to mitigate these [(**D**) to (**F**)], and the conceptual ideas of CIDER mechanisms that protect the tracer from relaxation sources [(**G**) to (**I**)]. Paramagnetic impurities (left column), chemical exchange (center column), and intermolecular collisions (right column) are prominent sources of relaxation (top row). Current mitigating approaches usually focus on removing these sources (middle row). The CIDER concept (bottom row) proposes a different mechanism: CIDER agents protect the target molecule by increasing the distance to relaxation sources (left and right) or slowing down rapid proton exchange (middle). The shielding effect of CIDER is illustrated schematically as circles surrounding the target molecule, mediated, e.g., by hydrogen bonding between agent and additive. The mechanism involving two hydrogen bonds shown in this figure is only exemplary: Fewer or more hydrogen bonds, as well as clustering (hydrotropic effects at high concentrations), may also be possible.

The typical sources of nuclear spin relaxation in the liquid state are paramagnetic impurities (Fig. 1A), chemical exchange (Fig. 1B), and intra- or intermolecular dipole-dipole interactions (Fig. 1C). Some of these effects can be beneficial, e.g., for clinical MRI, where gadolinium-based contrast agents are used to boost the magnetic resonance (MR) signal of water protons by accelerating the recovery of longitudinal polarization (*14*). In contrast, the diagnostic benefit of HMR would drastically increase if there was a way to prolong the nuclear spin relaxation of the hyperpolarized tracers.

There are two common approaches to address intramolecular dipole-dipole relaxation. One is to use long-lived spin states (LLS) (*15*). Unfortunately, although LLS can be less sensitive to intermolecular interactions than typical magnetization (*16*), this approach is not widely applicable, as it requires at least a pair of strongly coupled spins (*17*, *18*) and is usually destroyed by intermolecular collisions, oxygen in solution (*19*), and paramagnetic impurities (*16*, *20*). Another common approach is the substitution of protons with deuterium (*21*, *22*), which is especially critical for quickly relaxing glucose carbons (*11*, *12*), or the substitution of ^14^N with ^15^N for the same reason (*23*). Intramolecular deuteration can substantially prolong the lifetime but does not affect external sources of relaxation; addressing these is more complicated.

The relaxing effect of paramagnetic impurities can be strongly reduced by adding chelating agents, radical scavengers, and filtering or quenching radicals (Fig. 1D) (*24*–*28*). However, it is difficult to account for all impurities as they differ in nature and are not always known. For example, EDTA, a chelating agent often used in dDNP experiments, is effective for anions but not cations (*29*) and may have other unwanted effects (e.g., on catalysis).

The fluctuating magnetic fields caused by intermolecular interactions can be ameliorated by reducing the dipolar moment of the solvent by deuteration (Fig. 1F) (*30*–*32*). Using pure D_2_O, however, is expensive and may affect metabolism (*33*, *34*).

Besides distant interactions, the effect of pH and proton chemical exchange on ^13^C polarization was reported for the hyperpolarized fumarate and succinate using parahydrogen-induced polarization (PHIP). The polarization of fumarate decreased from about 10% at a basic pH of 14 to about 1% at an acidic pH of 3 (*35*). In previous studies, however, ^13^C polarization of succinate >10% was retained during transfer (from polarizer to MR through low field) at low pH and buffering at high field, but all polarization was lost if the sample was neutralized before transfer at low fields (prepared by PHIP at 2 mT and pH 3) (*30*, *36*, *37*). Notably, these studies did not report basic pH during sample transfer, preventing a direct comparison between them.

Chemical exchange–driven relaxation is efficient when the exchange rate is much faster than the Larmor precession frequency (*38*). Hence, the chemical exchange can be slowed down to suppress this relaxation source, e.g., by cooling the solution or changing pH (Fig. 1E) (*9*). However, neither extreme pH nor extreme temperatures are commonly suited for an experimental setup or biological application (*39*).

These effects render the use of some hyperpolarized tracers difficult to impossible. For example, recently, we found that the ^15^N nuclear spin of 1-^15^N-nicotinamide (1-^15^N-NAM) can be successfully hyperpolarized with dDNP in the solid state to a high degree. However, dissolving and transferring the sample often annihilated the polarization so that no signal was observed (*39*). The effect was caused by the fact that, at neutral pH, the lifetime of 1-^15^N-NAM hyperpolarization was in the order of 7 s at 1 T, and about 1 s at few millitesla. Consequently, most or all polarization was lost during the transfer to the measuring site. We found, too, that hyperpolarization was maintained at basic pH, where the exchange is suppressed.

Here, we report on a previously unrecognized approach to address rapid relaxation, particularly in low magnetic fields. We found that some additives drastically increased the *T*_1_ of a dissolved hyperpolarized or thermally polarized tracer, especially at low fields and near p*K*_a_. These additives included biomolecules such as urea, NAM, glycerol, and glycerol-based dendrons; all had hydrogen-bond donor sites, indicating a remarkable protective mechanism of chemically induced deceleration of nuclear spin relaxation (CIDER) (Fig. 1, G to I). Magnetic field–cycling (MFC) experiments between 7.8 μT and 9.4 T with thermally polarized samples showed that CIDER can triple low-field *T*_1_ without changes in pH. For hyperpolarization, the effect drastically reduced the loss in polarization during transfer from polarizer to MRI. This CIDER effect was demonstrated with hyperpolarized 1-^15^N-NAM, ^13^C-NAM, ^15^N-pyridine, ^15^N-pyrimidine, and ^15^N-metronidazole (MNZ). For example, much or all hyperpolarization of 1-^15^N-NAM was lost when the neutral pH sample was transferred from DNP to MRI/nuclear magnetic resonance (NMR) without CIDER. With CIDER, however, the polarization of 1-^15^N-NAM was quantified to polarization (*P*) = 30% at the time of detection, after dissolution and transfer (at a slightly basic pH) and to *P* = 4% at a neutral pH.

## RESULTS AND DISCUSSION

### dDNP of 1-^15^N-NAM in the presence of NAM, urea, glycerol, and dendrons as CIDER agents

**Fig. 2.**
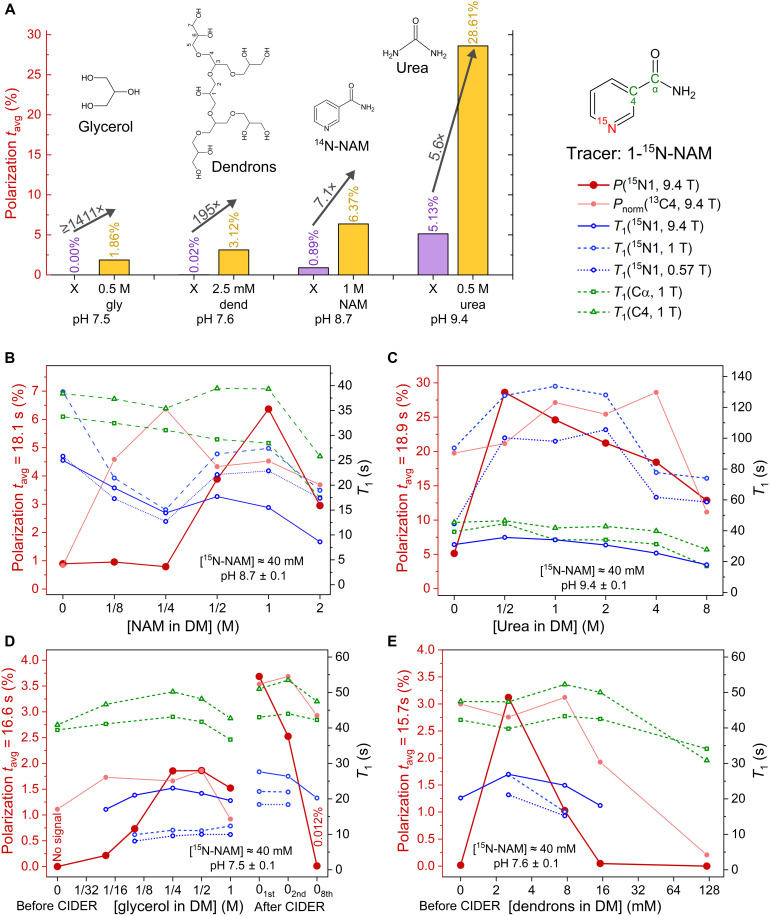
Illustration of CIDER concentration effects on relaxation and hyperpolarization of 1-^15^N-NAM. CIDER additives increase polarization (**A**) and affect high-field *T*_1_. Adding n.a. NAM (**B**), urea (**C**), glycerol (**D**), or dendrons (**E**) to the DM in dDNP experiments increased the liquid-state polarization of 1-^15^N-NAM drastically, noted by the arrows and “enhancement” values with/without the additives (detected after transfer through low fields). For example, the polarization (*P*) increased from 5 to 30% when 0.5 M urea was added (B). ^13^C hyperpolarized signal intensities were scaled to the highest ^15^N *P* for visual representation. For NAM, urea, and glycerol, the largest effect was observed when the ratio of NH─ or OH─ groups to the tracer was ~25 (fig. S3). The impact of the additives on the high-field (0.57 to 9.4 T) *T*_1_ of ^15^N and ^13^C varied; often, *T*_1_ was found to increase and later decrease with additive concentration. As the high-field *T*_1_ does not account for the stark differences in observed polarization, relaxation during sample transfer appears to be the key element affected by the additives as suggested by Fig. 4. The data acquired after the [glycerol] = 1 M experiment with a neat DM showed a remarkable increase in *P*, even though no CIDER agent was added (D). Note the uniformly strong increase in *P* from <0.1 to 5% as a result of pH increase from 7.5 to 9.4 [first data point in (A) to (D)], indicating chemical exchange as a source of relaxation as described before (*39*). To reduce the effect of varying transfer times, we report the estimated polarization for an average transfer time, *t*_avg_ [e.g., indicated as “polarization at *t*_avg_ = 18.1 s” in (A)] (text S10), except for *P*(^13^C). The pH at [CIDER] = 0 was adjusted with NaOH to remain constant throughout each series, and Cα and C4 of NAM were detected at n.a. of ^13^C.

To test CIDER, we polarized 1-^15^N-NAM with DNP and performed the dissolution with media at different pH of 7.5, 8.7, and 9.4 (Fig. 2). After transferring the sample in 15 to 18 s to the NMR, we observed no polarization when the pH 7.5 dissolution medium (DM) was used, 0.9% with pH 8.7 DM, and 5.1% with pH 9.4 DM. These findings are in agreement with our previous observations, where no polarization was observed at neutral pH and some at basic pH (*39*).

We repeated the experiments with different amounts of CIDER additives at natural isotopic abundance (n.a.) in the DM (n.a. NAM, urea, or glycerol). In all cases, a robust and reproducible polarization was observed (Fig. 2A): At neutral pH, the polarization rose from not observable (below 0.01% polarization) to 1.9% by adding glycerol (0.5 M; Fig. 2). At high pH, CIDER was found to improve the polarization further from 0.9 to 6.4% at pH 8.4 by adding 1 M n.a. NAM (Fig. 2) and from about 5 to 29% at pH 9.4 by adding 0.5 M n.a. urea (Fig. 2). Although each additive affected the polarization differently, all showed the largest effect when the ratio of the additive’s NH_2_─ or OH─ groups to the investigated tracer was ~25 (fig. S3).

Across all 25 experiments shown in Fig. 2D, the coefficient of variation of the solid-state signal was only 10.6%, indicating sufficiently high reproducibility. However, the observed liquid-state polarization varied several-fold (from undetectable to 30%). These data indicate that the variability in solid-state polarization is much less pronounced than the effect of CIDER additives in the DM. These data suggest some protective interaction of the additive with either relaxation-causing constituents or the tracer itself.

When using CIDER, we observed a move of the 1-^15^N-NAM resonance to higher chemical shift upon addition of CIDER additives (see figs. S1 and S2), about 0.35 parts per million (ppm)/M, 0.15 ppm/M, and −1.3 ppm/M for NAM, urea, and glycerol, respectively. This is consistent with the shift observed in the case of hydrogen bonding (*40*, *41*).

When we conducted experiments without CIDER additives after the experiment with 1 M glycerol, we found that the polarization increased yet again from 1.5 to 3.7% in the first experiment, followed by a steady decrease to about 0.012% in the eighth repetition (see Fig. 2D and table S6). This may reflect additional residual effects in the dissolution system, such as persistent glycerol coating on internal surfaces or sustainable cleaning of trace paramagnetic impurities in the system. While we adhere to manufacturer-recommended cleaning protocols—flushing the tubing and reservoirs three times with 10 ml of deionized water, followed by drying with air between experiments—this long-term behavior suggests that even extensive washing may not eliminate such effects immediately.

We also quantified *T*_1_ by monitoring the decay of the hyperpolarized ^15^N1, ^13^Cα, and ^13^C4 at 0.57, 1, and 9.4 T. The addition of 0.5 M urea, for example, more than doubled ^15^N1 *T*_1_ at 0.57 T from 44 to 100 s. *T*_1_ was also increased for the carbons ^13^Cα and ^13^C4. At high field, however, *T*_1_ decreased at very high concentrations of CIDER agents. The slowed molecular tumbling of NAM due to interactions with the additives and an increased chemical shift anisotropy (CSA), as previously predicted in the case of hydrogen bonding (*40*), can rationalize this effect.

We also investigated ^15^N *T*_1_ and *T*_2_ of thermally polarized 1-^15^N-NAM and low concentrations of glycerol at neutral pH at 9.4 T (fig. S2). Here, we found that *T*_1_ varied slightly, ranging from 14.9 to 15.1 s for 0 to 100 mM glycerol. In contrast, *T*_2_ was found to change by ~20% from 145 to 192 ms under the same conditions measured with Hahn echoes. For small molecules in the extreme narrowing regime, one typically expects *T*_1_ ≈ *T*_2_ due to rapid molecular tumbling. However, this was not observed in our case. We attribute the difference to a dominant relaxation mechanism—chemical exchange (*42*)—and paramagnetic impurities, which operate on a longer correlation time scale than molecular tumbling. As a result, *T*_1_ remains much longer at high fields compared to *T*_2_ (*43*).

As glycerol was found to be an effective CIDER additive, we synthesized and tested glycerol-based dendrons (Fig. 2D; nine OH groups per molecule) (*44*–*46*). When a molecular concentration of ~128 mM (~1.2 M [OH]) was used in the DM, the hyperpolarized ^15^N signal of 40 mM 1-^15^N-NAM vanished after the transfer, and the Cα and C4 ^13^C signal was strongly reduced. When the dendrons were diluted to 2.5 mM (~20 mM [OH]), an intense ^15^N polarization of 3.7% was observed, and *T*_1_ increased to 26.9 s (at 9.4 T), higher than observed after the addition of glycerol (all at pH 7.6). Currently, the detailed effect is under more thorough investigation.

It should be noted that NAM exhibits CIDER properties and, thus, can act on itself. Consequently, all NAM experiments contained 40 mM of labeled 1-^15^N-NAM, even if there were no CIDER agents in the DM.

### Effects of pH, D_2_O, and CIDER agents on ^15^N polarization

**Fig. 3.**
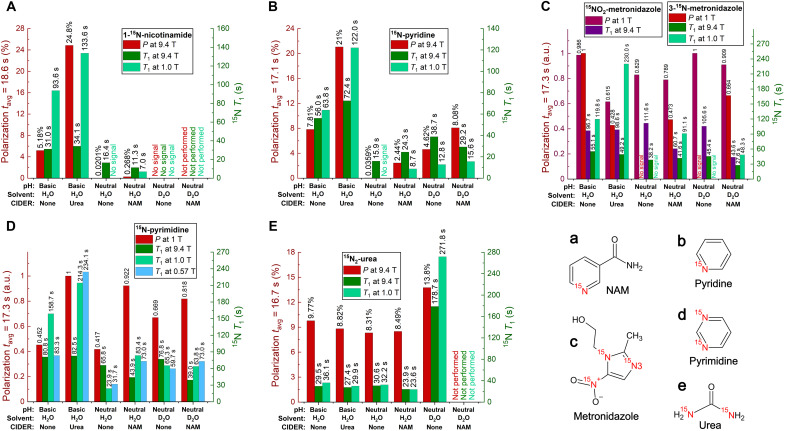
Impact of CIDER, pH, and solvent on hyperpolarization and relaxation of five molecules. Effect of CIDER, pH, and solvents on ^15^N polarization and *T*_1_ of 1-^15^N-NAM (**A**), ^15^N-pyridine (**B**), n.a. MNZ (**C**), n.a. pyrimidine (**D**), and ^15^N_2_-urea (**E**). 1-^15^N-NAM (a), ^15^N-pyridine (b), 3-^15^N-MNZ (c), and ^15^N-pyrimidine (d) showed a substantial increase in retained polarization at neutral and basic pH when CIDER agents, urea, or NAM were added. The polarization of urea (e) and ^15^NO_2_ of MNZ (c) was less affected. Urea’s *T*_1_ and polarization increased much when H_2_O was replaced by D_2_O. The DM consisted of 300 mg of Trizma buffer and 10 mg of EDTA dissolved in 50 ml of water (H_2_O or D_2_O) with different additives: H_2_O with 65 mg of NaOH (basic-H_2_O-none, pH 9.4 ± 0.02), H_2_O with 1 M n.a. urea (basic-H_2_O-urea, pH 9.4 ± 0.08), H_2_O (neutral-H_2_O-none, pH 7.5 ± 0.07), D_2_O (neutral-D_2_O-none, pH 7.6 ± 0.11), H_2_O with 1 M n.a. NAM (neutral-H_2_O-NAM, pH 7.5 ± 0.03), and D_2_O with 1 M n.a. NAM (neutral-D_2_O-NAM, pH 7.5 ± 0.02). Stock solutions (4 ml) were taken for each experiment. The polarization values were normalized to the *t*_avg_ [e.g., indicated as “polarization at *t*_avg_ = 18.6 s” in (A)] in each series as described in text S10. a.u., arbitrary units.

To assess the applicability of the CIDER effect, we conducted a series of hyperpolarization experiments using enriched 1-^15^N-NAM, ^15^N-pyridine, ^15^N_2_-urea, and MNZ and pyrimidine at n.a. of ^15^N. Each compound was measured at a basic pH of 9.4 and a neutral pH of 7.5, with and without the addition of n.a. urea or NAM as CIDER agents (Fig. 3). Details of the experimental parameters and measurements presented graphically in Figs. 2 and 3 are given in tables S3 to S13.

The addition of 1 M urea at basic pH increased *T*_1_ and enhanced the retained dDNP polarization for 1-^15^N-NAM, ^15^N-pyridine, 3-^15^N-MNZ, and ^15^N-pyrimidine but did not show beneficial effects on ^15^NO_2_-MNZ and ^15^N_2_-urea. For instance, while pyridine *T*_1_ was previously reported to be 41 s at pH 8.4 and 9.4 T (*47*), we observed an increased value of 72.4 s at 9.4 T and a staggering value of 122 s at 1 T and over 200 s for pyrimidine using 1 M urea as a CIDER additive at pH 9.4.

Similar but much stronger effects were observed at a neutral pH of 7.5 when 1 M NAM was added to the DM. The retained polarization increased more than 10-fold for 1-^15^N-NAM and almost 70-fold for ^15^N-pyridine. Furthermore, no signal was detected for 3-^15^N-MNZ at neutral pH without CIDER, while adding NAM resulted in a detectable signal. At basic pH, CIDER agents negatively affected the observable polarization of 3-^15^N-MNZ. The maximum observed polarizations for NAM and pyridine were 24.8% (Fig. 3A) and 21% (Fig. 3B), respectively, and considerably higher than previously reported (*39*). The fact that, among the three ^15^N sites in MNZ, only 3-^15^N was strongly affected by the CIDER agents and that this site is the only one capable of acting as a hydrogen bond acceptor supports the hypothesis that hydrogen bond formation plays a key role in the CIDER mechanism.

Urea was not affected much by the tested CIDER agents (urea and NAM); here, the largest effect on the observed polarization was observed when we substituted H_2_O with D_2_O, almost doubling the polarization to 13.8% (Fig. 3E). Of note, optimizing the urea sample composition using trehalose as a glassing agent and D_2_O as a DM, a maximum polarization of 19% was achieved with an average ± standard deviation of 16.5 ± 2.2% based on three measurements, which is higher than values reported in the literature between 2.3 and 7.8% (*31*, *32*, *48*, *49*). Sugars have previously been used successfully as glassing agents and as viable alternatives to glycerol and dimethyl sulfoxide (DMSO) (*50*–*52*).

An important observation was that replacing H_2_O with D_2_O increased the CIDER-enhanced polarization and *T*_1_ of pyridine even more, from 0.06% in H_2_O to 2.4% with 1 M NAM in H_2_O, to 4.6% in D_2_O, and finally to 8.1% in D_2_O with 1 M NAM. Similarly, the *T*_1_ at 1 T consistently increased under these conditions.

### CIDER on thermally polarized samples (*T*_1_ of 1-^15^N-NAM at different magnetic fields)

We constructed an MFC system to measure ^15^N *T*_1_ relaxation dispersion between 7.8 μT and 9.4 T, thereby validating the CIDER effect with thermally polarized samples. MFC experiments were conducted using a home-built flexible-rod shuttling system; previously, similar systems with solid rods have been presented (*53*, *54*). The system is described in detail elsewhere (*55*). The feasibility of studying nuclear magnetic-field relaxation dispersion (NMRD) using MFC is readily established (*56*) and represents one critical step in optimizing hyperpolarization experiments (*57*, *58*).

**Fig. 4.**
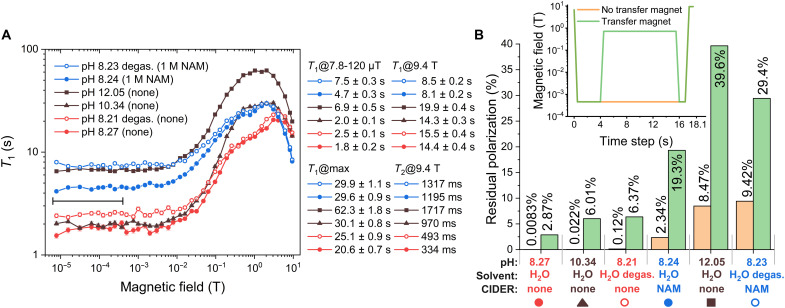
Impact of CIDER, pH, and degassing on nuclear magnetic relaxation dispersion of 1-^15^N-NAM and retained hyperpolarization. Effect of pH, CIDER, and degassing on *T*_1_ of 1-^15^N-NAM at 10^−5^ to 9.4 T (**A**) and estimated effect on polarization after 18-s transfer (**B**). MFC experiments were conducted to measure *T*_1_ of 100 mM 1-^15^N-NAM at pH 8.27 (red, ${\text{NAM}}^{\text{pH}\;8}$ ), pH 8.24 with 1 M NAM (blue, ${\text{NAM}}_{\text{CIDER}}^{\text{pH}\;8}$ ), pH 10.34 (dark brown triangles, ${\text{NAM}}^{\text{pH}\;10}$ ), and pH 12.05 (dark brown squares, ${\text{NAM}}^{\text{pH}\;12}$ ). Two samples were compared in normal and degassed (hollow spheres) states. For *B* < 10^−2^ T, *T*_1_s were relatively constant, with CIDER providing two- to threefold longer *T*_1_ than without it at the same pH. Degassing increased *T*_1_ by up to 60% and when combined with CIDER, the effects were additive. For 10^−2^ to 10^−1^ T, *T*_1_ increased to 20 to 63 s, with the shortest for ${\text{NAM}}^{\text{pH}\;8}$ and the longest for ${\text{NAM}}^{\text{pH}\;12}$ . From fields >3 T, all *T*_1_ declined to 8 to 20 s, with the shortest for ${\text{NAM}}_{\text{CIDER}}^{\text{pH}\;8}$ and the longest for ${\text{NAM}}^{\text{pH}\;12}$ . Using these data and assuming simplified field trajectories for the transfer of hyperpolarized samples (B, insert), we found that using CIDER increases the level of polarization after transfer without (and with) transfer magnet by 281× (7×) compared to ${\text{NAM}}^{\text{pH}\;8}$ and by 106× (3.2×) compared to ${\text{NAM}}^{\text{pH}\;10}$ . The ${\text{NAM}}^{\text{pH}\;12}$ increased the retained polarization further by 3.5× (2.1×) compared to ${\text{NAM}}_{\text{CIDER}}^{\text{pH}\;8}$ . This reflects the results that were observed in Figs. 2 and 3. *T*_2_ has been quantified by Hahn echo. The samples were prepared similarly to the dissolved DNP sample. pH was adjusted using NaOH. The standard deviation for each *T*_1_ value was plotted in the graph but was typically smaller than the symbol. Examples of *T*_1_ inversion-recovery kinetics are in fig. S4.

Here, we conducted 39 experiments for each sample using a *T*_1_-inversion-recovery sequence where most of the recovery occurred at low-field *B*_LF_. The sequences consisted of inverting thermal longitudinal magnetization of ^15^N at *B*_0_ = 9.4 T, followed by shuttling to low-field *B*_LF_ in ~1 s, waiting for a variable time Δ*t*, moving to *B*_0_ in about 1 s, exciting with 90°, and acquiring free induction decay. For each *B*_LF_, signals for 19 different Δ*t* values were acquired. We investigated the effect of CIDER, pH, and degassing on the *T*_1_ of 100 mM 1-^15^N-NAM for six samples (Fig. 4):

1) ${\text{NAM}}^{\text{pH}\;8}$ , the neutral pH without CIDER additives: Expected fast relaxation at low fields (neutral pH–control case; Fig. 1, A to C);

2) ${\text{NAM}}_{\text{degas}}^{\text{pH}\;8}$ , degassed ${\text{NAM}}^{\text{pH}\;8}$ sample: Expected prolonged relaxation at low fields (neutral pH–degas case; Fig. 1D);

3) ${\text{NAM}}^{\text{pH}\;10}$ , a more basic pH of 10.34 without CIDER additives: Expected prolonged relaxation (slightly pH–tuned case; Fig. 1E);

4) ${\text{NAM}}^{\text{pH}\;12}$ , basic pH of 12.05 without CIDER additives: Expected even more prolonged relaxation (strongly pH–tuned case; Fig. 1E);

5) ${\text{NAM}}_{\text{CIDER}}^{\text{pH}\;8}$ , identical to ${\text{NAM}}^{\text{pH}\;8}$ (1) but with CIDER (neutral pH of 8.24 with 1 M ^14^N-NAM as CIDER additive): Expected prolonged relaxation (CIDER case; Fig. 1, G to I);

6) ${\text{NAM}}_{\text{CIDER degas}}^{\text{pH}\;8}$ , degassed ${\text{NAM}}_{\text{CIDER}}^{\text{pH}\;8}$ : Expected even more prolonged relaxation (CIDER-degas case; Fig. 1, G to I);

As expected, the ^15^N relaxation strongly depends on *B*_LF_, pH, degassing, and the addition of CIDER (Fig. 4A). One may distinguish four typical relaxation regimes:

1) The ^15^N-NAM *T*_1_ was relatively constant for 10^−5^ to 10^−2^ T and about 200 to 300% longer for ${\text{NAM}}_{\text{CIDER}}^{\text{pH}\;8}$ (average across 7.8 to 120 μT, 4.7 ± 0.3 s) than for ${\text{NAM}}^{\text{pH}\;8}$ and ${\text{NAM}}^{\text{pH}\;10}$ (average across 7.8 to 120 μT, 1.8 ± 0.2 and 2.0 ± 0.1 s). Still, ${\text{NAM}}^{\text{pH}\;12}$ was about 50% longer than ${\text{NAM}}_{\text{CIDER}}^{\text{pH}\;8}$ , explaining the best-attained polarization at basic pH (Fig. 3A).

2) For *B*_LF_ = 10^−2^ to 1 T, all *T*_1_ increased, with the largest *T*_1_ changes for ${\text{NAM}}^{\text{pH}\;10}$ (from 2 to 30 s) to the level of ${\text{NAM}}_{\text{CIDER}}^{\text{pH}\;8}$.

3) At medium fields of 1 to 3 T, ^15^N *T*_1_ flattened. *T*_1_s of ${\text{NAM}}_{\text{CIDER}}^{\text{pH}\;8}$ and ${\text{NAM}}^{\text{pH}\;10}$ (29.6 and 30.1 s) were about 45% longer than that of ${\text{NAM}}^{\text{pH}\;8}$ (20.6 s). The ${\text{NAM}}^{\text{pH}\;12}$ experienced the longest *T*_1_ of 62.3 s.

4) For *B*_LF_ > 3 T, all *T*_1_ decreased. At 9.4 T, the highest field investigated, the *T*_1_ of ${\text{NAM}}^{\text{pH}\;12}$ was the longest (19.9 s) and for ${\text{NAM}}_{\text{CIDER}}^{\text{pH}\;8}$ was the shortest (8.1 s).

These data show a strong effect of CIDER additives on *T*_1_, especially at low fields, where CIDER additives tripled *T*_1_, and slowing exchange (by increasing pH) slightly improved or quadrupled *T*_1_, depending on pH. At the same time, the increase of *T*_1_ relaxation time in high fields supports the hypothesis of the interaction of NAM with the CIDER agents in high fields, where chemical exchange and paramagnetic impurity-induced relaxation are not effective, while CIDER interactions slightly accelerate relaxation.

Degassing of the DM led to an increase of low-field *T*_1_ by 39 and 60% for the ${\text{NAM}}_{\text{degas}}^{\text{pH}\;8}$ and ${\text{NAM}}_{\text{CIDER degas}}^{\text{pH}\;8}$ samples compared to their nondegassed counterpart, respectively; the impact from CIDER is substantially higher than from degassing: low-field *T*_1_ of ${\text{NAM}}_{\text{CIDER}}^{\text{pH}\;8}$ was almost double of ${\text{NAM}}_{\text{degas}}^{\text{pH}\;8}$ . Notably, the relaxation contribution of oxygen at low fields was reduced by 49% when 1 M NAM was added (fig. S6). This showcases the ability of CIDER additives to protect from paramagnetic impurities such as O_2_ or free radicals; paramagnetic relaxation is most efficient in the low-field regime (figs. S4 and S5). However, at medium to high fields, almost no difference was observed between the degassed and nondegassed samples: 8 and 5% for the samples without and with CIDER at 9.4 T, respectively.

Using the *T*_1_ data, we estimated the polarization loss during the transfer from the dDNP to the NMR (Fig. 4B). We assumed two field trajectories with and without a holding magnet (Fig. 4B, insert) (*55*): Without the transfer magnet, (i) the field was dropped at *t* = 0 from 6.7 T (in the dDNP) to 0.46 mT in 0.5 s, followed by (ii) a constant 0.46 mT for *t* = 0.5 s to *t* = 16.9 s and (iii) an increase in 0.6 s to 9.4 T for detection. With the transfer magnet, the trajectory was the same, only that a field of 0.7 T was applied between *t* = 4.5 s and *t* = 15.5 s. The transfer field was chosen to be 0.46 mT (based on the measured values of 15.3 mT at maximum and 28 μT at minimum in between DNP and NMR), and the transfer time was assumed to be the average delivery time of Fig. 2B).

The gain in *T*_1_ induced by CIDER increased the polarization retained after 18 s of transfer by a factor of 281× without the transfer magnet or by 6.7× with the transfer magnet ( ${\text{NAM}}^{\text{pH}\;8}$ versus ${\text{NAM}}_{\text{CIDER}}^{\text{pH}\;8}$ ). Using a more basic pH 10.3 increased the retained polarization by 2.7× (without transfer magnet) or 2.1× (with transfer magnet), and for pH 12.1 by 1018× (without transfer magnet) or 13.8×. The highest increase without a transfer magnet was estimated for the ${\text{NAM}}_{\text{CIDER degas}}^{\text{pH}\;8}$ sample by 1135×.

In addition, we measured *T*_2_ at 9.4 T using Hahn echoes. We found that increasing the pH from 8.3 to 10.3 and 12.1 resulted in a 2.5- or 4.4-fold increase in *T*_2_ (334, 970, and 1717 ms). Adding CIDER, ${\text{NAM}}_{\text{CIDER}}^{\text{pH}\;8}$ , tripled *T*_2_ (to 1195 ms) compared to ${\text{NAM}}^{\text{pH}\;8}$ , correlating with the observed increase in the low-field *T*_1_. Degassing of samples also extended low-field *T*_1_ and high-field *T*_2_. The Pearson correlation coefficient between *T*_2_ values measured at high fields and *T*_1_ values measured at low fields for the examined six samples was about 0.85 (Fig. 4).

### Discussing the CIDER mechanism

#### Physical aspects

In general, relaxation is caused by several overlapping effects. Three relevant effects in the context of this study are chemical exchange, paramagnetic impurities, and CSA, all of which are affected by molecular motion. Chemical exchange relaxation is most effective at low magnetic fields where the exchange rate constant is much larger than the Larmor frequency, making the mechanism highly field dependent. For instance, if a field is chosen such that the Larmor frequency is much faster than the exchange rate, the contribution of this effect to the overall relaxation diminishes. CSA, in contrast, becomes more effective at high fields.

The dramatic loss of polarization and acceleration of *T*_1_ at lower fields of ^15^N nuclei near exchanging hydrogens (Figs. 2 and 3) appears to be attributed to the rapid chemical exchange (fig. S7) (*38*) and combined with relaxation from paramagnetic impurities (fig. S5) (*20*, *59*, *60*) including oxygen (fig. S6). For example, in MNZ, the 3-^15^N nucleus, which can be protonated, was affected by pH and CIDER much more strongly than the other ^15^N nuclei. Shifting the pH away from p*K*_a_ reduces relaxation by rapid chemical exchange at low fields (Figs. 1E and 4), thereby preserving more polarization (Figs. 2 and 3). Such an effect was observed for NAM (*39*) with a p*K*_a_ of 3.35 (*47*), fumarate (*35*) with a p*K*_a_ of 2.86 to 3.02 (*61*), and succinate (*30*, *36*, *37*).

We consider that CIDER additives reversibly bind to the agents investigated, thereby reducing exchange, shielding from paramagnetic impurities, and slowing down molecular tumbling. Suppressing exchange and shielding from paramagnetic impurities have different effects on relaxation and depend on the magnetic field: *T*_1_ is prolonged at low fields due to slowed down exchange and shielding from paramagnetic impurities, and accelerated at high fields due to slowed down molecular tumbling and increased CSA (Fig. 4). The NMRDs with degassed samples align with the hypothesis of CIDER additives shielding from paramagnetic impurities. We observed that the benefits of CIDER and degassing are additive; however, degassing is less relevant in case of CIDER, indicating a larger average distance between the tracer and the paramagnetic impurities (fig. S6).

The observed chemical shift data of 1-^15^N-NAM (fig. S1) demonstrated a proportional change in chemical shift with increasing CIDER concentration. This is consistent with the hydrogen bonding effect between the tracer and the CIDER additive—the decreased electron density on the 1-^15^N of NAM leads to deshielding and, thus, a higher chemical shift (*40*, *62*). However, the chemical shift move depends strongly on the hydrogen bond partners and can also lead to a lower chemical shift (*41*, *63*). The stronger change in shift per molar of added NAM (0.33) compared to urea (0.15) suggests stronger hydrogen bonding in the case of NAM (*62*, *64*).

Low-field *T*_1_ is highly relevant for transferring hyperpolarized samples. The CIDER mechanism leads to better polarization preservation at low fields (supported with NMRD data, Fig. 4), despite a reduced high-field relaxation rate, which follows our dDNP observations: The CIDER low-to-medium field effect is most relevant for retaining polarization. In high fields, the impaired motion from the higher molecule density and intermolecular interactions in ${\text{NAM}}_{\text{CIDER}}^{\text{pH}\;8}$ leads to increased CSA and intramolecular dipole-dipole relaxation contributions and, hence, reduced *T*_1_; however, this has very little relevance for the retained polarization after transfer through fields of 0.5 to 1000 mT. This observation also explains why the high-field *T*_1_ (Figs. 2 and 3) does not correlate with the retained polarization. For example, while CIDER reduces the high-field *T*_1_, the retained polarization is higher by one to two orders of magnitude, depending on the conditions (Figs. 2 to 4).

In a series of samples compared—${\text{NAM}}^{\text{pH}\;8}$ , ${\text{NAM}}_{\text{degas}}^{\text{pH}\;8}$ , ${\text{NAM}}_{\text{CIDER}}^{\text{pH}\;8}$ , ${\text{NAM}}_{\text{CIDER degas}}^{\text{pH}\;8}$ , ${\text{NAM}}^{\text{pH}\;10}$ , and ${\text{NAM}}^{\text{pH}\;12}$—the Pearson correlation coefficient between *T*_2_ values measured at 9.4 T and *T*_1_ at low fields (average across 7.8 to 120 μT) was found to be about 0.85 (Fig. 4), indicating a strong correlation. Hence, *R*_2_ relaxometry may be a valuable tool for studying the binding effects of the CIDER additives on the tracer (*65*, *66*). The reason is that *T*_1_ and *T*_2_ both have a field-dependent spectral density function $\sim \frac{\tau_{C}}{1+{\left(\omega \tau_{C}\right)}^{2}}$ , while *T*_2_ has an additional field-independent contribution, $\sim \tau_{C}$ , where $\tau_{C}$ is the correlation time, and ω is a Larmor precession frequency. Since, in the case of a high field, $\omega \tau_{C}\gg 1$ for chemical exchange, the contribution to *T*_1_ diminishes, while it is still present for *T*_2_ because of the second contribution. At low fields, however, $\omega \tau_{C}\ll 1$ ; hence, the impact on *T*_1_ is large and similar to the one on *T*_2_.

Moreover, we observed accelerated relaxation and reduced polarization when an excessive concentration of CIDER agents was used, which, in extreme cases, such as high concentrations of dendrons, completely destroys the polarization. This suggests an interaction between CIDER agents and the tracer, as hypothesized. Notably, the Cα relaxation of NAM is typically faster than that of C4; however, with a high concentration of dendrons, this trend is reversed. This reversal is likely because the mobility of the coordinated pyridine ring decelerates much faster than that of the labile amide carbon (Fig. 2D).

The trityl radical was not removed upon dissolution. Although radical removal via acidification, filtration, and subsequent neutralization is sometimes used (*28*), in our case, this approach led to a complete loss of polarization when we attempted it for 1-^15^N-NAM. This is likely because the radical removal was performed at low magnetic fields of a few millitesla and requires additional time, causing relaxation. Complete polarization loss is expected considering the rapid relaxation at low fields (Fig. 4), which was estimated before to be around 2.5 s at low fields (*39*). In addition, the concentration of trityl radical after dissolution was only ~30 μM, and varying this amount (e.g., halving) showed a negligible effect on the preserved polarization [see (*39*)]. Moreover, observation of the rapid *T*_1_ for NAM at pH 8 and 10 without CIDER confirms that the traces of trityl and oxygen (Fig. 4) are not the primary reasons for the observed rapid polarization losses in dDNP experiments.

Solution viscosity is another critical factor influencing relaxation, as it linearly increases the molecular correlation time and thereby accelerates relaxation [see equation 2.43 of (*67*)]. For instance, the addition of 0.25 M glycerol increases the viscosity by ~8%, and by ~40% at 1 M (*68*, *69*). While such changes in viscosity contribute to the observed decrease in *T*_1_ time at high magnetic fields (see Fig. 2D), they are insufficient to account for the pronounced polarization preservation at low fields. Therefore, additional mechanisms beyond solution viscosity effects play a critical role in the observed relaxation behavior.

To conclude, hydrogen bonding between the CIDER additive and the tracer provides a coherent explanation for the observed effects (Fig. 1). The interaction shifts the ^15^N chemical shift downfield (high chemical shifts), consistent with the withdrawal of electron density. At low and intermediate magnetic fields, relaxation is reduced due to shielding from paramagnetic impurities and an increased distance between dipole-dipole partners, as well as impaired chemical exchange of hydrogen. At the same time, hydrogen bonding leads to slower molecular tumbling and increased CSA, which increases relaxation, especially at high fields. The resulting *T*_1_ and observed polarization behavior thus reflects a balance: At optimal CIDER concentrations, shielding effects dominate and polarization is preserved, while at high concentrations or fields, the relaxation enhancement from restricted motion outweighs the benefits.

#### Chemistry aspects

All the identified potent CIDER agents, NAM, urea, glycerin, and dendrons, act as hydrogen bond donors, which may form hydrogen bonds with the tracers or sources of relaxation.

The strongest hydrogen-bond donors are strong acids, which concomitantly lower the solution’s pH to unphysiological values and increase chemical exchange. Thus, hydrogen bond donor agents based on N─H bonds (instead of O─H), which are intrinsically less acidic, are promising CIDER agents. Electron-withdrawing substituents at the N─H group increase the N─H donor strength. Therefore, urea and NAM may be efficient CIDER agents because the C═O groups are electron withdrawing, increasing the H-bond donor strength, while they affect pH only a little. Moreover, urea can donate up to two hydrogen bonds via its two N─H donor groups (and accept via C═O), which is possibly the reason why a lower concentration is needed compared to NAM (*70*). Glycerol may also form multiple hydrogen bonds with its three OH groups.

We compared the ratio of the CIDER agent concentration to tracer concentration, for which the maximum amount of polarization was preserved (fig. S3). More detailed analysis at different tracer concentrations is needed to investigate this hypothesis. Previously, we found that the concentration of 1-^15^N-NAM and trityl does not influence high-field *T*_1_, while the amount of DM is more important [see fig. S5 and (*39*)]. Therefore, this may suggest that chelation effects contribute to the complexation of trace paramagnetic metals such as Fe^3+^ or other impurities. However, no additional testing for sample purity was performed, and all reagents were used as received. Glycerol was shown to exhibit chelating properties, especially in its deprotonated glycerolate forms (*71*). Therefore, chelating effects in the case of glycerol (and possibly of dendrons as relatives to glycerol) cannot be ruled out; the possibility of chelating in addition to CIDER properties even appears reasonable. Since EDTA was present in all experiments and slight variations in its concentration showed no notable effect, its contribution to the observed phenomenon appears limited. For a list of hydrogen-bond donors and acceptors, see (*72*).

### Issues in using CIDER agents

The DM is superheated to about 200°C before dissolution. Under these conditions, urea decomposes, producing ammonia, oxides of nitrogen, cyanuric acid, cyanic acid, biuret, and carbon dioxide (*73*). Isocyanates such as cyanuric acid react with water to unstable carbamic acid, decomposing into CO_2_ and ammonia. Consequently, some ammonia and CO_2_ are created when using urea as a CIDER agent in the dDNP, resulting in a slightly basic pH of ~9.5 (p*K*_a_ of ammonia). However, most urea remains intact during the brief heating needed before the dissolution. This amount was estimated to be over 97.1%, since 8 M urea was observed with a signal-to-noise ratio (SNR) of 34.7, and no other compounds next to NAM and urea were visible when looking at the samples of Fig. 2C. Adjusting the pressure, temperature, and heating time of the DM can help control these effects. While NAM does not encounter such issues, it appears to be a less efficient CIDER agent than urea and glycerol. In addition, direct ammonia testing as a potential CIDER agent did not improve polarization, suggesting that intact urea is responsible for the observed CIDER effect.

The thermal stability of CIDER agents plays an important role in dDNP. For example, common hydrogen bond donors such as thiourea or cysteine are unsuitable CIDER agents in dDNP due to their decomposition to hydrogen sulfide during superheating. Similarly, the potential agent glucose was found to caramelize when the DM was superheated, leaving a lasting olfactory impression. Other applications of CIDER, which do not require high temperatures, however, will not suffer from this issue.

Thus, a challenge in using CIDER in biomedical hyperpolarized MRI lies in identifying an efficient, stable molecule that does not affect in vitro or in vivo applications. In this regard, glycerin emerges as one of the best candidates, exhibiting a strong CIDER effect without observable detriments.

We preliminarily tested other CIDER compounds, such as 2,2′-bipyridine and ammonia, and other tracers such as pyruvate and glucose with CIDER agents. Detailed results are provided in text S11. In the case of pyruvate, arguably the most widely used molecule in hyperpolarization, no substantial changes in retained polarization were observed in the presence of 1 M 1-^14^N-NAM or urea. This outcome is consistent with the already long low-field *T*_1_ of 1-^13^C-pyruvate (typically >30 s) (*55*), which may limit the observable benefit from CIDER agents compared to tracers with inherently shorter *T*_1_, such as 1-^15^N-NAM under neutral pH. However, altering the CIDER conditions may provide a benefit even for slower relaxing molecules such as pyruvate.

#### CIDER for HMR

Undoubtedly, *T*_1_ is one of the most prominent and valuable features of magnetic resonance. Contrast agents to reduce *T*_1_ of target molecules are widely used in medicine and analysis. Agents that prolong *T*_1_ without directly targeting paramagnetic impurities, however, were hitherto unknown. The CIDER effect described here was particularly effective in prolonging *T*_1_ of ^15^N nuclei in proximity to exchanging protons at low fields and at pH near p*K*_a_, ^13^C nuclei were less susceptible to the effect. This feature is particularly relevant for biomedical hyperpolarization: While some molecules or nuclei, such as ^15^N-urea or ^15^NO_2_-MNZ, retain the hyperpolarization during transfer, others, such as 1-^15^N-NAM or 3-^15^N-MNZ, lose their polarization at low fields within a few seconds [MNZ and NAM are tracers with the potential biomedical application: The antibiotic MNZ was recently tested as a hypoxia probe (*74*), while NAM can be used as a tracer in inflammatory bowel diseases (*75*)]. CIDER addresses this issue. The data showed that adding CIDER agents before transfer drastically increased the polarization observed after transfer through low magnetic fields (Fig. 2A). Once the sample is transferred to the application site, e.g., for in vivo administration, the field is usually high enough (>1 T) so that the low-field relaxation effects are less effective (Fig. 3). Thus, a dilution of the CIDER agents at high field, after administration, does not present a problem. However, given the accelerated relaxation of hyperpolarization in the blood due to paramagnetic particles such as hemoglobin (*31*), a second generation of CIDER agents may be designed in the future to address this challenge too.

Similar effects of swift relaxation induced by rapid exchange at low magnetic fields were observed for some ^13^C spins (*35*, *36*). Where appropriate, CIDER agents are expected to improve performance and redefine the standard for the preparation of hyperpolarization. Prolonged ^13^C *T*_1_ of NAM when using CIDER agents (Fig. 2) also indicates this.

Although shorter transfer times are possible with specialized magnetic tunnels and automated systems (*76*, *77*), our ~20-s transfer of the hyperpolarized sample using Halbach arrays with >0.5 T magnetic fields (*39*) to the measuring site mimics a more clinically realistic scenario in which hyperpolarized material is transferred manually between separate rooms. Application of a handheld electromagnet for the transfer of hyperpolarized samples was also demonstrated (*78*, *79*); however, the typical field is below 10 mT, which is insufficient for moving 1-^15^N-NAM relaxation from the low-field regime with rapid relaxation to higher fields with much slower relaxation (Fig. 4A). The CIDER effect on hyperpolarized tracers, with the conditions presented here, thus reflects its performance under real-world conditions rather than idealized laboratory settings; when more rapid transfer is used, the benefit of such agents should be less prominent.

CIDER dramatically increased the lifetime of longitudinal polarization of suitable molecules at low fields and in the presence of chemical exchange. The CIDER effect was used to enhance the observable polarization level of several tracers by orders of magnitude, enabling 30% ^15^N hyperpolarization of NAM in solution, an essential mark for developing previously unexplored metabolic tracers.

A high observable polarization is paramount for any application in MR, as it determines the SNR and the sensitivity limit. Prolonging *T*_1_ and preserving more polarization at the time of detection via CIDER may be key to establishing additional tracers and applications of nuclear spin hyperpolarization. Although MFC provided insights into the underlying mechanisms, and the experimental evidence for this effect is robust, a more detailed analysis and understanding of the CIDER mechanism are desirable. The drastic impact of CIDER on spin properties is not limited to the scope of hyperpolarization but can also be used to study other phenomena, such as the weak molecular interactions between molecules in liquid states.

## MATERIALS AND METHODS

### Chemicals

#### Sample preparation for dDNP

Samples for dDNP were prepared by mixing a substrate with trityl radical (AH111501, Polarize) in deionized water with trehalose (90210, CAS: 6138-23-4, Sigma-Aldrich) (*50*–*52*). As a substrate, we used in-house synthesized 1-^15^N-NAM according to (*80*), ^15^N-pyridine (48183, Sigma-Aldrich), MNZ (M1547, Sigma-Aldrich), pyrimidine (131695, Sigma-Aldrich), and ^15^N_2_-urea (316830, Sigma-Aldrich).

1) NAM sample: 130 mg of water and 60 mg of trehalose, 100 mg of 1-^15^N-NAM, and 10.5 mg of trityl radical, resulting in a sample volume of 225 μl. The resulting trityl radical and 1-^15^N-NAM concentrations were around 29 mM and 3.6 M.

2) Pyridine sample: 100 mg of water and 48 mg of trehalose, 68 mg of ^15^N-pyridine, and 8.1 mg of trityl radical, resulting in a sample volume of 185 μl. The resulting trityl radical and ^15^N-pyridine concentrations were around 27 mM and 4.6 M.

3) MNZ sample: 300 mg of DMSO, 77 mg of MNZ, and 11.4 mg of trityl radical, resulting in a sample volume of 335 μl. The resulting trityl radical and MNZ concentrations were around 21.3 mM and 1.34 M.

4) Pyrimidine sample: 49 mg of trehalose, 108 mg of pyrimidine, and 5.8 mg of trityl radical, resulting in a sample volume of 144 μl. The resulting trityl radical and pyrimidine concentrations were around 25 mM and 9.3 M.

5) Urea sample: 130 mg of water, 104 mg of trehalose, 77 mg of urea, and 8.3 mg of trityl radical, resulting in a sample volume of 265 μl. The resulting trityl radical and urea concentrations were around 19.6 mM and 4.7 M.

#### Sample preparation for MFC

The samples were prepared using a solution mimicking the hyperpolarized sample: 100 mM 1-^15^N-NAM were added to a solution of 40.3 mM Trizma pre-set crystals, 0.27 mM EDTA, and 263 μM (non-CIDER samples, ${\text{NAM}}^{\text{pH}\;8}$ , ${\text{NAM}}^{\text{pH}\;10}$ , and ${\text{NAM}}^{\text{pH}\;12}$ ) or 289 μM (CIDER sample, ${\text{NAM}}_{\text{CIDER}}^{\text{pH}\;8}$ ) trityl radical. The pH was adjusted to the desired value with NaOH.

When prepared, the solution was stored at −24°C. Before use, the vial was warmed to room temperature and vortexed for 2 min. The typical sample size was 30 mg for pyridine and 50 mg for the others. Degassed samples were prepared using three freeze-pump-thaw cycles, followed by flame-sealing of the NMR tube to prevent reoxygenation.

#### ^15^N-nicotinamide

1-^15^N-NAM was synthesized in a two-step reaction from NAM (72340, CAS: 98-92-0, Sigma-Aldrich) via the Zincke salt followed by nitrogen exchange with ^15^NH_4_Cl (299251, CAS: 39466-62-1, Sigma-Aldrich). In the first step, the Zincke salt of NAM was formed with 1-chloro-2,4-dinitrobenzene (237329, CAS: 97-00-7, Sigma-Aldrich) in DMSO (*80*). The resulting compound was a slightly yellow powder, which was subsequently reacted with ^15^NH_4_Cl to obtain 1-^15^N-NAM as a white powder. In some cases, there was a yellow tint from the presence of 2,4-dinitroaniline after the chromatographic workup, which could not be separated chromatographically. In these cases, we propose to make an additional purification step with activated charcoal before the purification via column chromatography or additional recrystallization in ethyl acetate after the column chromatography. The best yield was 40% for 1-^15^N-NAM with 91 ± 2% ^15^N enrichment. The synthesis is detailed in (*39*).

#### Dissolution medium

The DM with a pH of 7.5 was prepared by mixing 300 mg of Trizma pre-set crystals (pH 7.6, average M = 149.0 g/mol; T7943, Sigma-Aldrich) and 10 mg of EDTA (11280, CAS: 9002-07-7, SERVA) in 50 ml of deionized water or 99.9% D_2_O (151882, Sigma-Aldrich) and stored at room temperature. Additional chemicals were added in some cases; if applicable, they are noted in the text. The substances included n.a. NAM at 0.125, 0.5, 1, and 2 M (72340, CAS: 98-92-0, Sigma-Aldrich); urea at 0.5, 1, 2, 4, and 8 M (2317, CAS: 57-13-6, Carl Roth); glycerol at 0.05, 0.1, 0.25, 0.5, and 1 M (CAS: 56-81-5, Carl Roth); dendrons at 2.5, 7.6, 15.3, and 122.0 mM (see text S8); 35 mM bipyridine (CDS018251, Sigma-Aldrich); and 1 M ammonia (S706093, CHEMSOLUTE), all at n.a. To achieve a DM with pH 8.5 and 9.4, around 50 and 65 mg, respectively, of NaOH (1355, CAS: 1310-73-2, ChemSolute) were added. pH values were adjusted to be used with different CIDER additives, such that for all concentrations of CIDER (even one much larger than the concentration of the buffer), pH stays the same; therefore, for urea as a CIDER, for example, we used pH 9.4.

### Hyperpolarization and NMR signal acquisition

#### dDNP

All dDNP experiments were performed using a cryogen-free dDNP system (SpinAligner) (*4*, *10*) at ~1.4 K and 6.7 T. A microwave (MW) frequency between 187.07 and 187.19 GHz with 10 to 45 mW power was used for polarization. The optimal MW frequency was calibrated for each sample batch by varying the MW frequency (*10*). For each DNP experiment, the indicated amount of the concentrate (typically 50 mg) was taken from the stock, filled into the sample cup, and lowered into the MW cavity at ~1.3 K. DNP was initiated by continuous wave irradiation at the optimized frequency and power. The buildup of the ^15^N polarization in the solid state was monitored every 5 to 15 min with a ^15^N radio frequency (rf) pulse of 3°. The flipping angle of the in-built NMR was calibrated to ^15^N prior to the experiments.

#### NMR and MRI

^15^N MR signals were acquired using two 1 T ^13^C and ^15^N benchtop NMR (Spinsolve Carbon and Nitrogen, Magritek), a 9.4 T wide bore NMR (WB400, Avance NEO, Bruker) with a 5-mm broadband fluorine observe (BBFO) double resonance probe , and a benchtop, 0.57 T, 10-mm MRI system (“magspec” magnet unit, “drive L” console unit, Pure Devices GmbH). No locking at 9.4 T was used during the hyperpolarization experiments.

#### Sample transfer

For transportation to 0.57 and 9.4 T measuring sites, Halbach magnets with field variation across the sample between 0.65 to 1.05 T and 0.54 to 0.89 T correspondingly were used as described before (*39*).

#### Temperature

The temperature settings of the NMR systems were as follows: 37°C for experiments at 9.4 T, 30°C for 0.57 T, and 28°C and 26°C for the magnet and probe box, respectively, at 1 T. After dissolution, the sample temperature is temporarily elevated and gradually equilibrates to the system temperature during the *T*_1_ measurement.

#### Magnetic field cycling

The MFC system is described in (*55*). Here, a T_1_ inversion-recovery (T1IR) sequence was used to probe the *T*_1_ at fields from 7.8 μT to 9.4 T for three different samples. The recovery curve was fit in Origin using a monoexponential decay function.

### Quantification

#### Signal enhancement

The signal enhancement ε was quantified with respect to the accumulated signal of the same sample in thermal equilibrium using (Eq. 1)

$$P^{\text{HP}}=P^{\text{TP}}\cdot \varepsilon =P^{\text{TP}}\cdot \frac{S^{\text{HP}}}{S^{\text{TP}}}\cdot \frac{{\text{NS}}_{\text{acq}}^{\text{TP}}}{{\text{NS}}_{\text{acq}}^{\text{HP}}}\cdot \frac{\text{sin}\left(\alpha^{\text{TP}}\right)}{\text{sin}\left(\alpha^{\text{HP}}\right)}\cdot \frac{RG^{\text{TP}}}{RG^{\text{HP}}}$$ (1)

where $P^{\text{TP}}$ is the polarization in thermal equilibrium, $S^{x}$ is the integral of the respective signal, ${\text{NS}}_{\text{acq}}^{x}$ is the number of accumulated spectra, $\alpha^{x}$ is the excitation angle, $RG^{x}$ is linear receiver gain for hyperpolarized (*x* = HP) and thermally polarized (*x* = TP) NMR spectra. Exemplary hyperpolarized spectra together with their corresponding thermal spectra (only in the case of ^15^N labeling) are given in the Supplementary Materials (figs. S11 to S15).

#### Thermally polarized liquid-state NMR

Thermally polarized liquid state ^15^N NMR spectra were acquired for ^15^N-labeled compounds with high-resolution NMR at 9.4 T, ^1^H decoupling, and the following acquisition parameters: number of accumulations ${\text{NS}}_{\text{acq}}^{\text{TP}}$ = 64, flip angle $\alpha^{\text{TP}}$ = 90°, repetition time TR = 170 s, and total experimental time of about 3 hours. A typical SNR of 20 was obtained. ^15^N signals were quantified by manual integration after manual phase correction, line broadening, and baseline correction (MestReNova 14.2.2, Mestrelab Research S.L.). The selected integration region around the hyperpolarized signal was ±3 ppm. The polarization was quantified only at 9.4 T.

When n.a. of ^15^N compounds were used (it is the case for MNZ and pyrimidine), we could not directly observe the signal at thermal equilibrium: Estimated required experimental time is 3 hours × 1/(0.00365)^2^ ~ 26 years to compensate for 0.365% ^15^N n.a.; therefore, instead of absolute polarization, relative signals were compared (Fig. 3, C and D). To compensate for different transfer times in different experiments, the polarization values (Figs. 2 and 3) were normalized to the average transfer time in each series, as detailed in text S10.

#### Liquid-state polarization decay

The decaying hyperpolarization was sampled with $\alpha^{\text{HP}}$ = 5° to 10° pulse every 3 to 6 s. To quantify the lifetime of hyperpolarization, $T_{1}^{\text{HP}}$ , a monoexponential decay function was fit to the data, yielding the apparent/observed constant $T_{1}^{\text{obs}}$ (Eq. 2)

$$S^{\text{obs}}\left(t\right)=S_{0}\cdot e^{-\frac{t}{T_{1}^{\text{obs}}}}$$ (2)

To obtain the longitudinal relaxation time *T*_1_, $T_{1}^{\text{obs}}$ was corrected in all cases, if not otherwise stated, considering the polarization consumed by the repetitive rf excitations with angle $\alpha^{\text{HP}}$ as (*10*)

$$T_{1}=T_{1}^{\text{obs}}{\left\{1+\frac{T_{1}^{\text{obs}}}{\text{TR}}\text{ln}\left[\text{cos}\left(\alpha^{\text{HP}}\right)\right]\right\}}^{-1}$$ (3)

## Supplementary Materials

### This PDF file includes:

Supplementary Text Figs. S1 to S15 Eqs. S1 and S2 Tables S1 to S13 References
