# Supplementary material for: Chemically induced deceleration of nuclear spin relaxation (CIDER) preserves hyperpolarization
Source: Sci Adv. 2025 Sep 12;11(37):eadx2316. doi: 10.1126/sciadv.adx2316 (PMC12429058; doi:10.1126/sciadv.adx2316)
Supplement: Supplementary file 1 — Supplementary Text Figs. S1 to S15 Eqs. S1 and S2 Tables S1 to S13 References [file sciadv.adx2316_sm.pdf]

Science Advances
AAAS

Supplementary Materials for

Chemically induced deceleration of nuclear spin relaxation (CIDER) preserves hyperpolarization

Josh P. Peters *et al.*

Corresponding author: Andrey N. Pravdivtsev, andrey.pravdivtsev@rad.uni-kiel.de

Sci. Adv. **11**, eadx2316 (2025)
DOI: 10.1126/sciadv.adx2316

This PDF file includes:

Supplementary Text
Figs. S1 to S15
Eqs. S1 and S2
Tables S1 to S13
References

Supplementary Text, Figures, and Tables

S1. ^{15}N Chemical shift of 1-^{15}N-NAM as a function of NAM and urea CIDER additive concentration

Due to the rapid relaxation of tracer hyperpolarization under certain conditions, it was necessary to measure the signal as soon as possible. Therefore, since it takes time to lock before the measurement can start, no locking was performed for the dDNP experiments. Additionally, we aimed to minimize any extraneous influences on our experiments, such as D_2O when it was not desired. Therefore, a 100% H_2O solution was used instead of adding 10-20% D_2O to lock the signal. The NMR spectrometer was typically locked and shimmed about 20 minutes before dDNP on the reference H_2O+D_2O sample, and subsequently, the lock was switched off. Hence, dDNP and subsequent thermal reference spectra were measured without active lock.

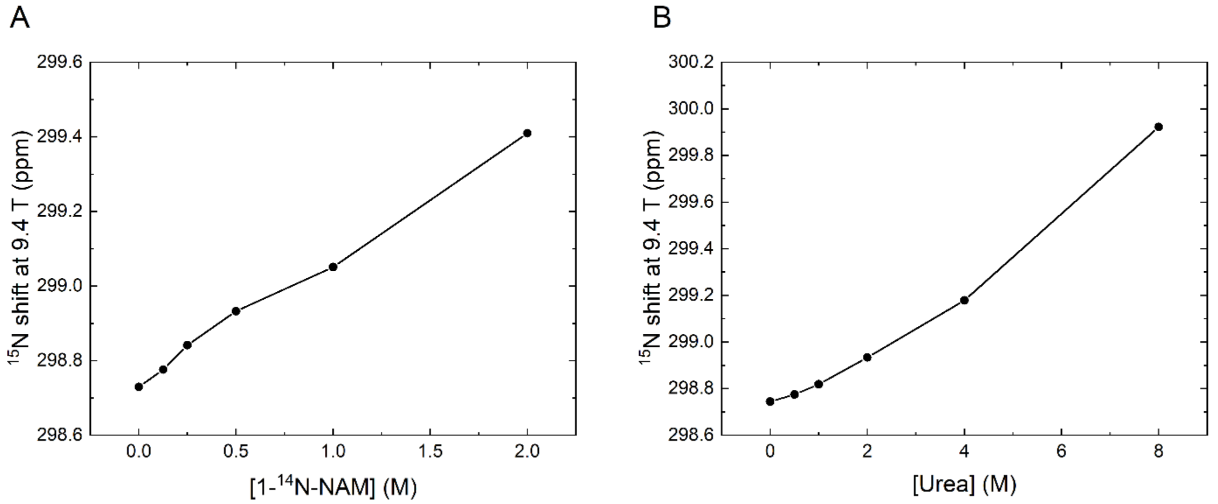

Figure S1: Effect of ^{14}N-NAM (A) and urea (B) concentrations as CIDER additives on the chemical shift of [1-^{15}N]NAM. The chemical shifts from thermal spectra following the experiments presented in **Fig. 2B** (NAM, A here) and **Fig. 2C** (urea, B here) have been analyzed and plotted in dependence on CIDER additive concentration. A clear correlation was observed between the chemical shift (ppm) and additive concentration, indicating the donation of a partial positive charge from the hydrogen bond donor (^{14}N-NAM or urea) to the tracer ([1-^{15}N]NAM), thereby de-shielding the [1-^{15}N] nucleus. The withdrawal of electron density from the nitrogen leads to a higher chemical shift value; this observation is consistent with the proposed mechanism in the manuscript. Fitting a linear curve to the experimental data revealed a slope of 0.33 ppm/M and 0.15 ppm/M for NAM and urea, respectively.

S2. <u>Chemical shift, T_1 and T_2 as a function of the concentration of CIDER agents</u>

We carried out an additional experiment to observe changes of the chemical shift under the influence of CIDER additives. Chemical shifts were not additionally calibrated, but the sample was locked to the D_2O signal as conventionally done. T_1 was measured using an inversion-recovery sequence with a repetition time of 154 s, the variable delay list was following:

For all concentrations in seconds: 0.1, 1, 2, 3, 4, 5, 6, 8, 10, 15, 30, 60

T_2 was measured using Hahn echo sequence with a single refocusing pulse with a repetition time of 102 s and the variable echo time (time between the first pulse and the start of the acquisition) was listed as follows:

For 0 mM in seconds: 0.002, 0.005, 0.01, 0.02, 0.05, 0.1, 0.2, 1, 2

For 100 and 200 mM in seconds: 0.002, 0.02, 0.05, 0.1, 0.2, 0.5, 1, 2, 4

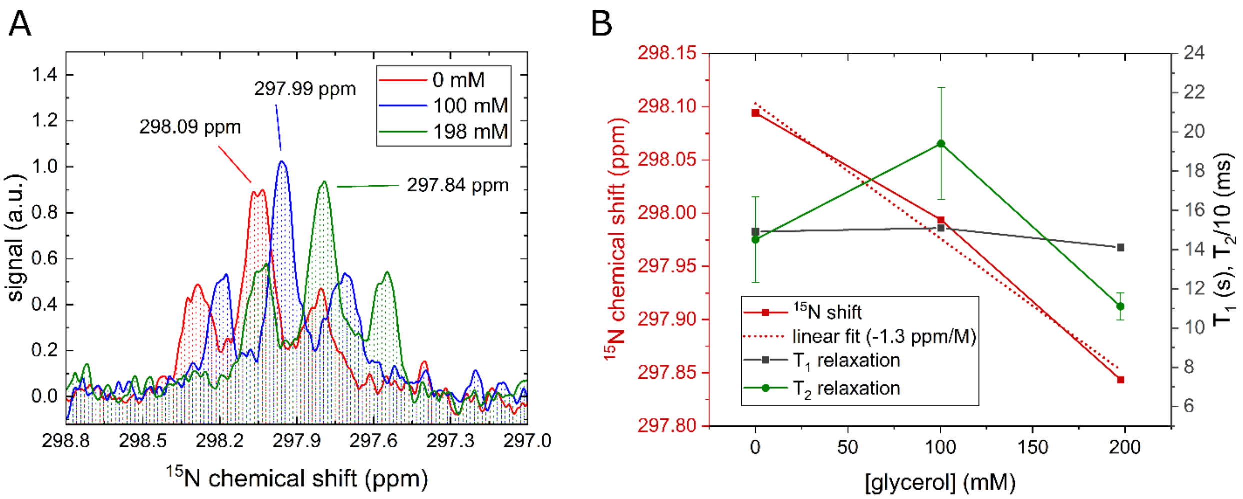

Figure S2: ^{15}N NMR spectra (left), chemical shifts, linear fit (right), and T_1, and T_2 values of ≈ 50 mM 1-^{15}N-NAM in solution with 0, 100, or 200 mM glycerol, acquired at neutral pH (pH 5.7), in thermal equilibrium at 9.4 T, 293 K measured with 90° pulse-excitation experiments and switched on D_2O lock. The ^{15}N resonance of thermally polarized NAM showed a triplet at ≈ 289.09 ppm, which shifted when glycerol was added to 297.99 (100 mM glycerol) and 297.84 ppm (198 mM glycerol), about 1.3 ppm/M as quantified with a linear fit (B). T_1 and T_2 of ^{15}N-NAM were found to change, too, from (T_1/T_2): 14.91 s / 144.8 ms, to 15.10 s / 191.8 ms, and 14.11 s / 107.8 ms, respectively. The polarization, detected at 9.4 T after transfer through the low field rose from <0.1% (0 glycerol) to 0.73% (100 mM glycerol) and 1.9 % (250 mM glycerol) (see **Figure 2** in the main manuscript).

Note that the trends of T_1/T_2(9.4 T) and observed hyperpolarization as a function of [glycerol] do not match, this is because main contribution for polarization losses is during transport of the sample at fields below 1 T, suggesting that relaxation at low fields does not follow this trend (see **Figure S3**).

Note, too, that T_2 at high field appears to be affected more by [glycerol] and thus the CIDER effect than T_1. When pH was shifted to the basic level of 13.3, the T_1 was 25.07 s, T_2 increased to 3.01 s, and the chemical shift to 289.13 ppm. All these are consistent with literature data on the high sensitivity of T_2 to the chemical exchange, even

in high fields,(*65*,*66*) and support of the hypothesis of CIDER interaction with the hyperpolarized tracer.

Details: The solution at neutral pH consisted of 47.8 mM 1-^{15}N-NAM in 547 uL H_2O and 60 uL D_2O, and 0, 100.4 mM, or 197.5 mM glycerol were added. At a high pH 47.9 mM 1-^{15}N-NAM in 552 uL H_2O and 60 uL D_2O with 3.22 mg NaOH to achieve a pH of 13.3 were used. Note, the measurements of T_1 and T_2 at high pH are weighted values for nicotinamide and nicotinic acid because of the long experiment duration and ongoing slow reaction from nicotinamide to nicotinic acid at such a high pH. For evaluation, we integrated signals of nicotinamide and nicotinic acid together.

S3. Impact of hydrogen bond donor groups of CIDER agents

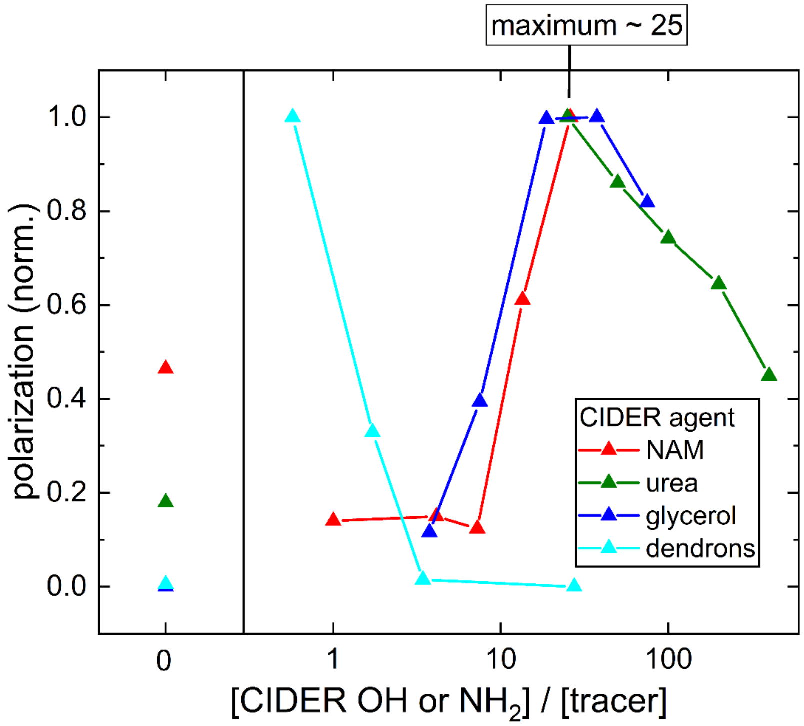

Figure S3: Normalized ^{15}N polarization as a function of the hydrogen-bridge-bond donor groups (HBDG) concentration of the added CIDER agents divided by the tracer concentration. While the maximum CIDER effect (i.e., polarization) was observed at different concentrations of each additive (**Figure 2**, main text), it occurred at similar concentrations of the hydrogen bond donor groups for NAM, urea, and glycerol: $[HBDG](P_{max}) \approx 25 \cdot [tracer]$.

We assume that the concentration needs to be this high so that the CIDER agent and tracer have a decently high chance of meeting in the solution. However, it should not be too high, such that the molecular motion is reduced by many additives clinging to a tracer, thus accelerating relaxation. The detrimental effects of high concentrations are also discussed in **Figure 2**. For dendrons, the approximate relationship $[HBDG](P_{max}) \approx 25 \cdot [tracer]$, observed for other CIDERs, does not hold. This deviation may stem from the dendrons' more complex and larger molecular structure, which likely impairs tracer mobility, reduces correlation time, and has high enough binding affinity even at lower concentrations. Consequently, while dendrons may initially act to preserve polarization as other CIDER agents, these secondary detrimental effects may dominate at higher concentrations, ultimately leading to faster relaxation and reduced polarization efficiency. Note that similar decline is observed for other molecules but at much higher concentrations.

S4. T1IR measurements of samples presented in Fig. 4

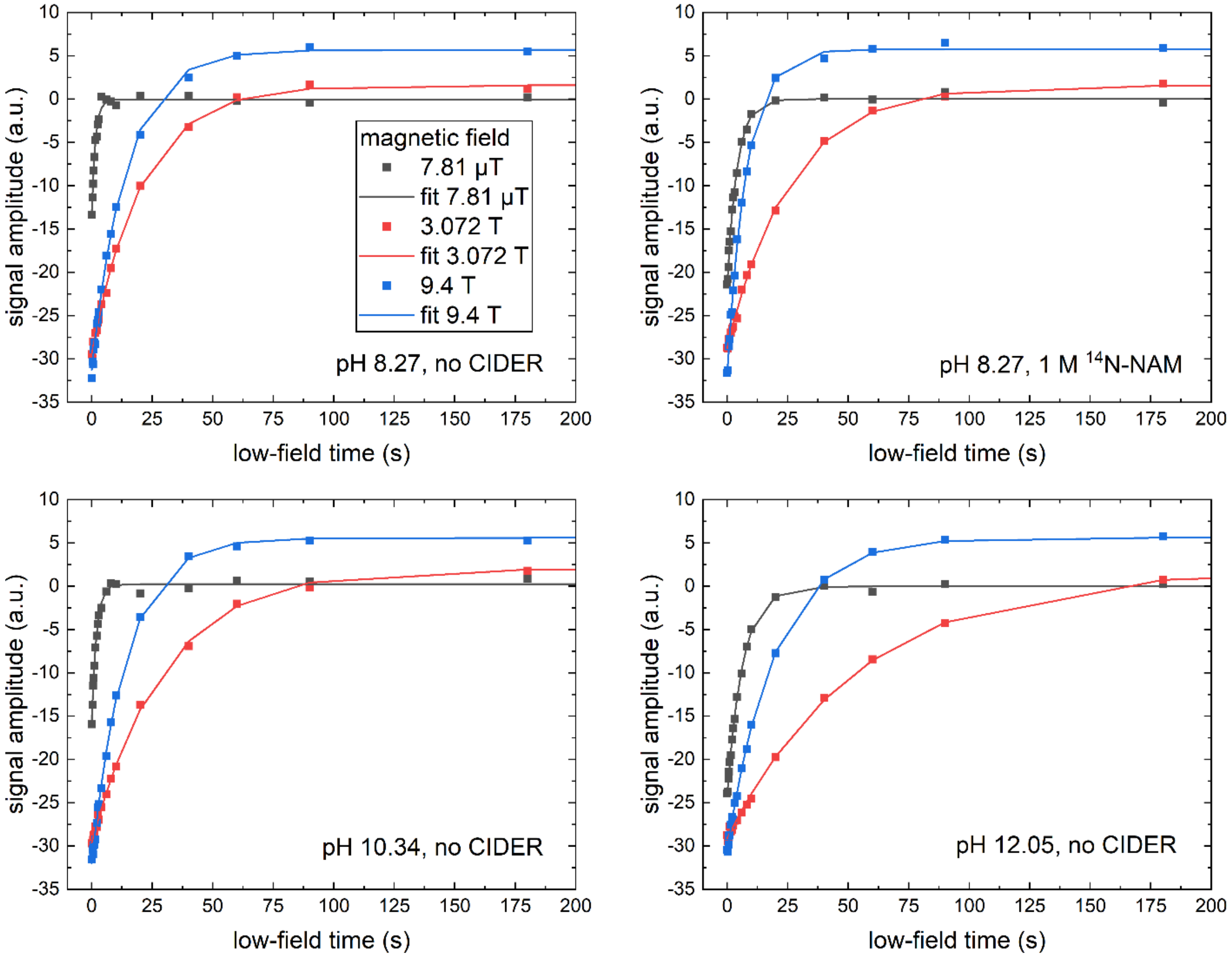

Figure S4: Three exemplary relaxation kinetics measured at different fields for the four samples presented in Fig. 4 of the main manuscript. (black) The lowest magnetic field of 7.8 µT, (blue) the field providing about the maximum T_1 at 3.1 T, and (red) the highest field of 9.4 T are shown here. The time was varied in 19 steps between 0 and 300 s using the following delay-list in seconds: 0, 0.25, 0.5, 0.75, 1, 1.5, 2, 2.5, 3, 4, 6, 8, 10, 20, 40, 60, 90, 180, 300. Each pseudo-2D experiment was performed for 39 desired fields and for the four different samples to obtain the kinetics shown here. The shortest T_1 at 7.8 µT is clearly visible with rapid decay and a lower signal for the start value of 0 s compared to the other fields due to relaxation losses. Also, slightly biexponential curve shapes can be observed for the lowest field at 5-10 s. However, a mono-exponential fit was found to be sufficient (lines with corresponding colors).

S5. <u>Relaxation induced by paramagnetic impurities</u>

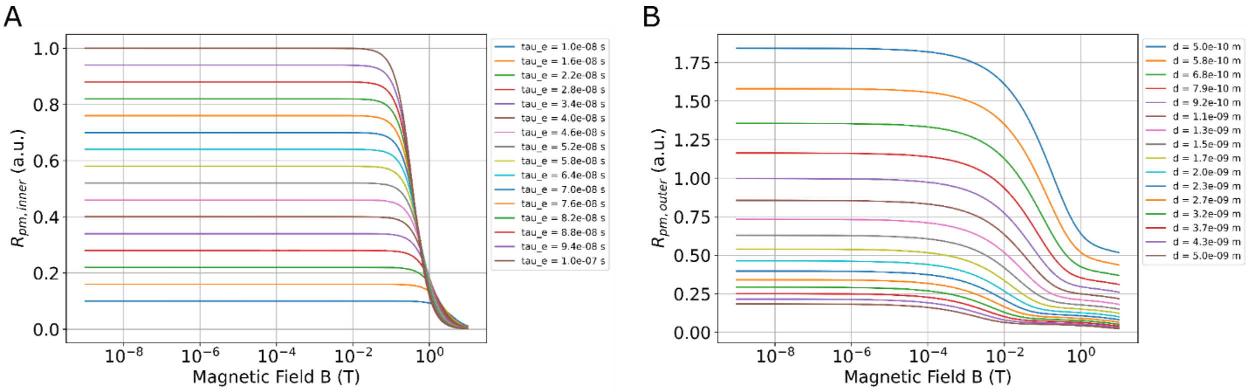

Figure S5: Inner (A) and outer sphere (B) contribution of paramagnetic impurities to the relaxation of ^{15}N tracers (R_{pm}) depending on the magnetic field(*59,60,20*)**.** As expected, relaxation induced by paramagnetic impurities is reduced in higher fields. Inner sphere relaxation becomes almost negligible at 9.4 T. Still, one can observe relaxation effects if, e.g., gadolinium is used in sufficiently high concentrations. In this case, the outer sphere relaxation is dominant. However, both contributions are much stronger in a low field (e.g., when transferring the sample from the polarizer to the spectrometer) than in high fields. This leads to accelerated relaxation even at low impurity concentrations, which aligns with the experimental data, where T_1 at a high field was still preserved when relaxation was rapid and below 1-3 s at a low field. This does not allow us to rule out paramagnetic impurities entirely from consideration.

Details: we used equation 3 for (A) and 4 for (B) from Ref. (*20*). The amplitudes for relaxation rates are given in arbitrary units. The purpose is a qualitative assessment of field dependencies. Relaxation rates were modeled using γ_e, γ_{15N}, a diffusion coefficient of $2.3 \cdot 10^{-9}$ m^2/s, the electron correlation times given in the legend of (A), and the distance of the closest approach of the molecule and radical given in the legend of (B).

S6. <u>Impact of degassing oxygen depending on CIDER additives</u>

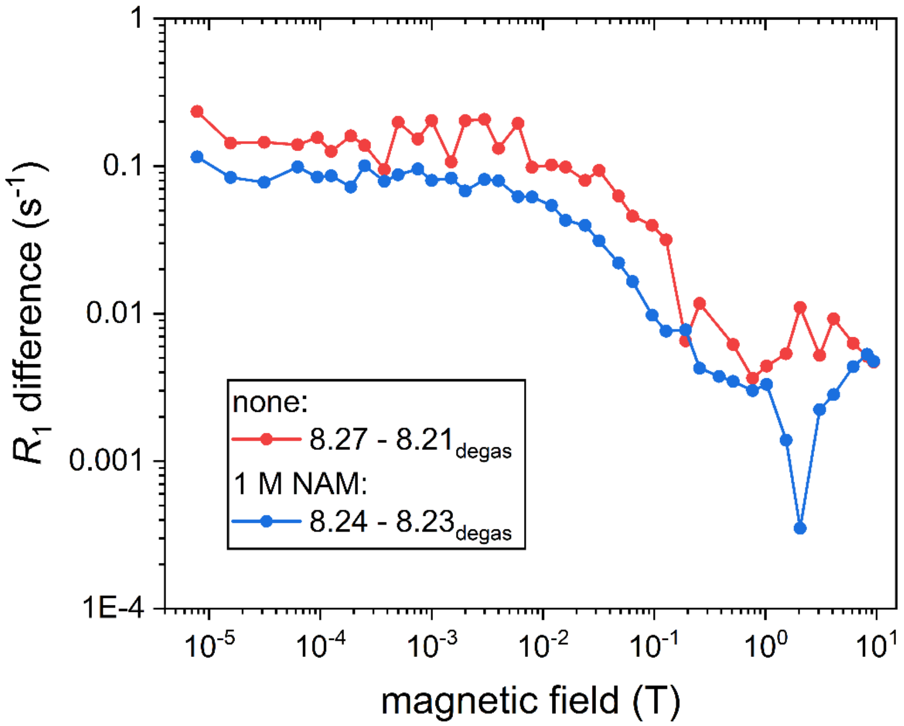

Figure S6: Effect of degassing on the neutral samples presented in Fig. 4 of the main text. Oxygen relaxivity was estimated as the difference in relaxation rates for $\mathrm{NAM}^{\mathrm{pH\ 8}}_{\mathrm{degas}}$ and $\mathrm{NAM}^{\mathrm{pH\ 8}}$ (none, red), as well as $\mathrm{NAM}^{\mathrm{pH\ 8}}_{\mathrm{CIDER\ degas}}$ and $\mathrm{NAM}^{\mathrm{pH\ 8}}_{\mathrm{CIDER}}$ (1 M NAM CIDER, blue). The stronger contribution of oxygen to the relaxation of the non-CIDER sample (red) can be observed compared to the 1 M NAM CIDER sample (blue). The contribution in the low field regime of 7.8 µT to 12 mT was reduced by about 49% with the addition of 1 M NAM. This was expected for the proposed mechanism of shielding of the tracer from both exchange and paramagnetic impurities, such as oxygen radicals or O_2, by the CIDER additives. The effect of oxygen removal diminishes for fields above ~0.2 T, as expected for paramagnetic impurities (**Fig. S4**). Note the log-scale on the y-axis.

S7. Effects of chemical exchange on relaxation

This section is reproduced from supporting information of Ref. (39) Peters et al. without modifications and is included here to complement the discussion of T_1 field dependences, especially in the context of paramagnetism-induced relaxation mechanisms discussed previously. We emphasize that this analysis, originally exemplified for scalar coupling relaxation of the second kind, leads to a requirement of an unrealistically large scalar coupling constant (here, ~5000 Hz was used only as an example to reach the very rapid relaxation at low fields). This value is clearly not physical and should be interpreted purely as a mathematical placeholder used to demonstrate that low-field relaxation can be significantly modulated by exchange processes. The general structure of relaxation rate expressions for scalar coupling and dipole–dipole interactions shares some similar dependence on spectral density functions. Thus, although this specific model is not quantitatively valid, it serves to qualitatively highlight that chemical exchange processes — including exchange-modulated dipole–dipole interactions or J-coupling — may plausibly contribute to the observed field-dependent T_1 behavior. A more realistic mechanism may involve dipolar interactions between, for example, ^{1}H and ^{15}N nuclei separated by ~1 Å. The fitting of the experimental magnetic field dependencies of relaxation could provide better estimates.

We observed the effect of pH on the relaxation properties of 1-^{15}N-NAM. When neutral-DM is used, no polarization was observed. Experimentally, we found that neutralization can only be achieved within the stray field and a short timespan between neutralization and start of measurement.

When a slowly relaxing nucleus, here ^{15}N, has J-coupling interaction with a rapidly relaxing or rapidly (chemically) exchanging nucleus X, then the contribution of the scalar coupling relaxation as a function of a magnetic field can be calculated as follows (Ref. 38, Chiavazza et al):

$$R_{1,\mathrm{SC}}^{15\mathrm{N-X}} = \frac{8\pi^2 J^2}{3} I_\mathrm{X}(I_\mathrm{X}+1)\frac{\tau_\mathrm{SC}}{1+(\omega_{15\mathrm{N}}-\omega_\mathrm{X})^2\tau_\mathrm{SC}^2} \qquad \text{(Eq. S1)}$$

Here J is the constant of ^{15}N-X scalar spin-spin coupling, I_X is the spin of nucleus X (½ for ^{1}H and 1 for ^{2}H), ω_{15N} is the Larmor angular precession frequency of coupled ^{15}N and X (^{1}H or ^{2}H here) spins respectively, and τ_SC is the correlation time characteristic of the scalar interaction. To estimate the T_1 value as a function of the magnetic field, we calculated the following value (**Figure S7**):

$$T_1 = \frac{1}{R_{1,\mathrm{SC}}^{15\mathrm{N-X}}+R_{10}} \qquad \text{(Eq. S2)}$$

where R_{10} is the primary (here field field-independent) relaxation rate from different sources of relaxation. A large drop in a lifetime is predicted in these cases at low magnetic fields (**Figure S6**). Systematic measurements of T_1 as a function of pH and magnetic field have the potential to reveal the nature of the ^{15}N hyperpolarization loss.

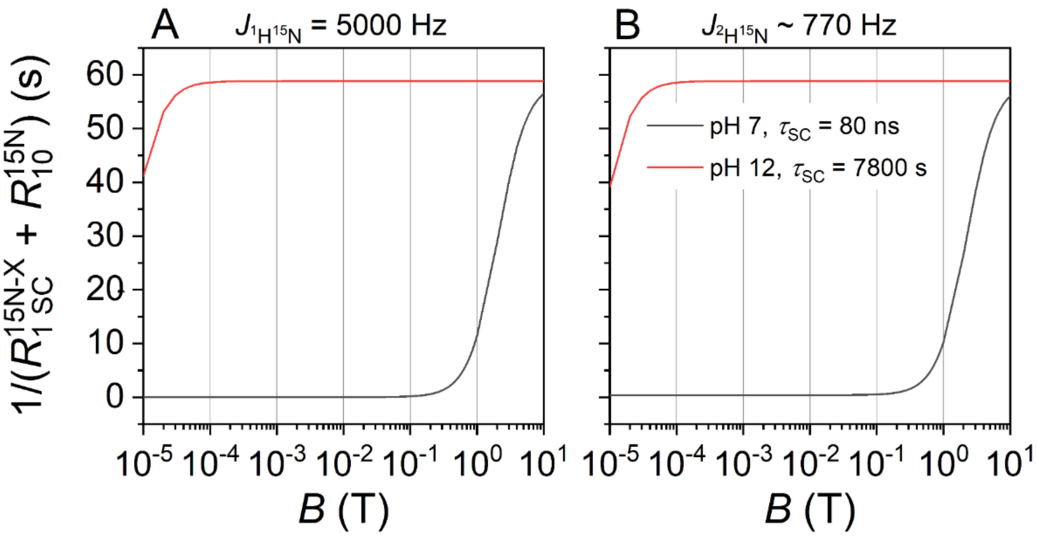

Figure S7. Effect of chemical exchange of quickly exchanging nucleus on the relaxation time of the slowly relaxing ^{15}N nucleus as a function of the magnetic field. The interaction with ^1H (A) and ^2H (B) were considered. $R_{1,SC}$ is calculated using Eq. S4 (Ref. (*38*), Chiavazza et al). The used parameters τ_{SC} = 80 ns (red), 7800 s (black). R_{10}^{15N} = 1/60 s^{-1}, ^1H-^{15}N interaction J_{1HN} = 5000 Hz, and ^2H-^{15}N interaction J_{2HN} = 5000/6.5 Hz were considered. This graph illustrates the effect of low magnetic fields on relaxation time in the presence of fast chemical exchange. Chemical exchanges of ^1H and ^2H from observations were compared, and simulations were adjusted accordingly: no signal was found for neutral H_2O or D_2O solvents, therefore, fast relaxation is expected in both cases at low fields. The correlation time was estimated as an average of NAM and HNAM lifetime: $\frac{1}{2}\left(\frac{1}{k_1[H]} + \frac{1}{k_2}\right)$ with $k_2 = 4 \cdot 10^{10}\ s^{-1}$ and $k_1 = k_2 10^{pKa}$. With these parameters, we can reproduce the fast relaxation at neutral pH and low magnetic fields, long relaxation time at basic pH and all magnetic fields, and relaxation time of around 10 s at neutral pH at 1 T. Such J value of 5 kHz is more in the order of dipol-dipol interaction instead of J-coupling. The estimated distance between ^1H and ^{15}N with a dipole-dipole interaction of J = 5 kHz can be estimated to be $r = \left(\frac{\mu_0 \gamma_{1H} \gamma_{15N} \hbar^2}{4\pi h J}\right)^{1/3}$ =1.37 Å.

S8. Dendrons

General Information

Commercially available solvents and reagents were used as received. Drying of THF was carried out on a Pure Solv MD-5 drying system from Innovative Technologies.
Column chromatography was carried out using 0.040-0.063 mm mesh silica gel from Merck. R_f values were determined by thin layer chromatography on Polygram® Sil G/UV$_{254}$ with 0.2 mm particle size from Macherey-Nagel. Identification of spots were carried out with Hanessian's stain: 0.2 % (m/V) $Ce(SO_4)$, 5 % (m/V) $(NH_4)_2Mo_3O_7$, 5 % (m/V) conc. H_2SO_4 in H_2O. After immersion, the silica gel plates were carefully dried using a heat gun. Substance spots then appear as blue spots.
NMR-spectroscopy was carried out on an AV 200 (^{1}H-NMR: 200 MHz), a DRX 500 (^{1}H NMR: 500 MHz, ^{13}C NMR: 125 MHz) or an AV 600 (^{1}H NMR: 600 MHz, ^{13}C NMR: 150 MHz) from Bruker. Further two-dimensional methods for assigning the peaks were used (COSY, HSQC, HMBC). Chemical shift is notated in ppm. ^{1}H NMR and ^{13}C NMR spectra were referenced to solvents from Deutero as follows:

Table S1: Solvent references in ^{1}H NMR and ^{13}C NMR(*81*)

solvent	^{1}H NMR shift [ppm]	^{13}C NMR shift [ppm]
acetone-d_6	2.05	29.84
$CDCl_3$	7.26	77.16
D_2O	4.79	21.03 (acetic acid)

Signal multiplicities were abbreviated as follows:

Table S2: Abbreviations of signal multiplicities

Abbreviation	Meaning
s	singlet
quint.	quintet
m	multiplet

IR-spectroscopy was carried out with a Perkin-Elmer 1600 Series FT-IR spectrometer with an A531-G Golden-Gate-Diamond-ATR-unit. The wave number range was 4000 cm^{-1} to 400 cm^{-1}. Signal intensities were noted as follows: vs (very strong), s (strong), m (medium), w (weak).

UV/vis spectroscopy was carried out on a Lambda 14 spectrometer from Perkin-Elmer.

Electron impact ionization (EI) was carried with an ionization energy of 70 eV on an AccuTOF GCv 4G mass spectrometer from Jeol. Electrospray ionization (ESI) high resolution (HR) spectra were measured with a Q Exactive™ Plus mass spectrometer with Hybrid Quadrupole Orbitrap™ from Thermo Fisher Scientific.

Synthetic Procedures

Dendrons were synthesized according to Wyszogrodzka *et al.* (Ref. (44)) starting with solketal. A double Williamson ether synthesis with methallyl dichloride yields dendron first generation. The resulting molecule must be prepared to build the next generation by undergoing ozonolysis and reduction to the corresponding alcohol afterward. The next steps for the next generation will then repeat themselves. After receiving the second generation of protected dendron, deprotection was carried out with acetic acid (**Fig. S8**).

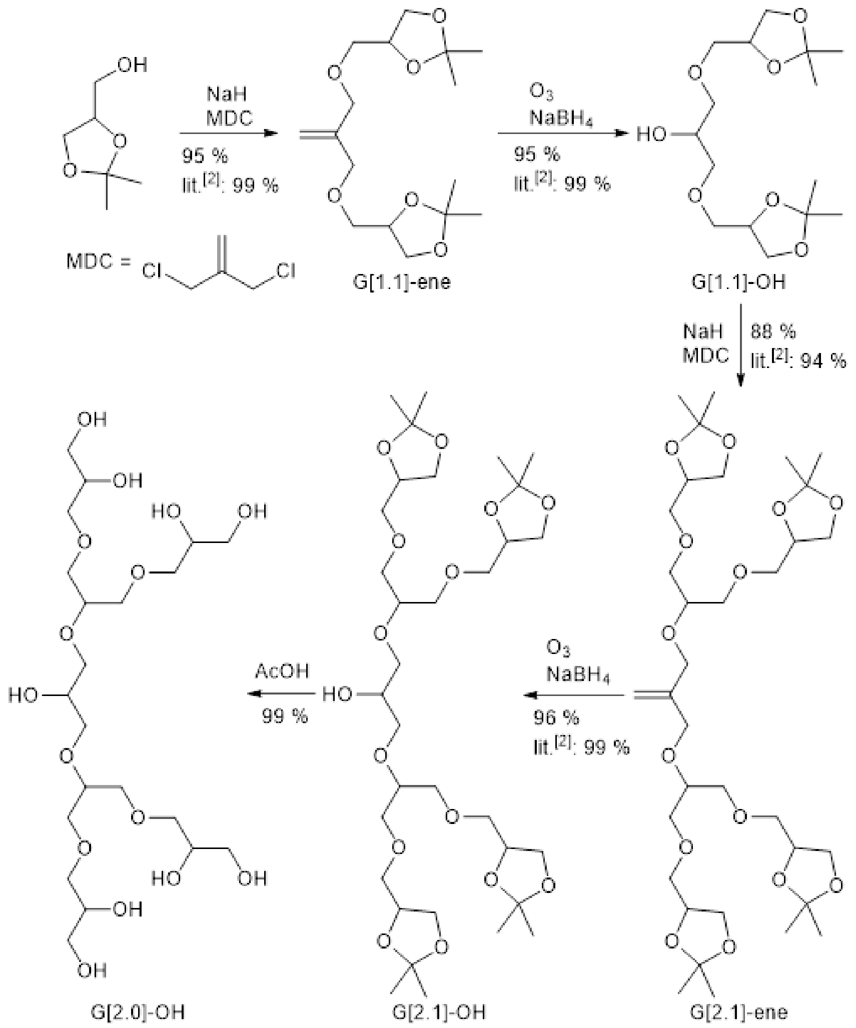

Figure S8. Repeating steps of Williamson ether synthesis and ozonolysis with reduction in order to build higher generations of glycerol dendrons.

General Procedure A: Synthesis of dendrons G[n.1]-ene

Under a nitrogen atmosphere 3-4 equivalents of sodium hydride were placed in 100-200 mL dry tetrahydrofuran. To the mixture 2 equivalents G[n-1.1]-OH were added dropwise and catalytic amounts of [15]-crown-[5] were added. After stirring for 1 h at 40 °C, 1 equivalent of methallyl dichloride and catalytic amounts of potassium iodide were added. Stirring continued under reflux for 1 d. The reaction was quenched by adding 100 mL water and the phases were separated. The aqueous phase was extracted with dichloromethane and the combined organic phase was dried over magnesium sulfate. The solvent was removed under reduced pressure and the crude product was purified by column chromatography on silica gel.

General Procedure B: Synthesis of dendrons G[n.1]-OH

G[n.1]-ene was dissolved in 100-200 mL dry methanol/dichloromethane 1:1 under a nitrogen atmosphere and cooled to -78 °C. Ozone bubbled through the solution until it turned blue. Excess of ozone was removed by bubbling nitrogen through the solution. The reaction mixture was treated with sodium borohydride and stirred for 16 h while allowing to warm to room temperature. Reaction was quenched with 100 mL water, extracted with dichloromethane and the organic phase was dried over magnesium sulfate. The solvent was removed under reduced pressure and yielded the corresponding G[n.1]-OH.

G[1.1]-ene

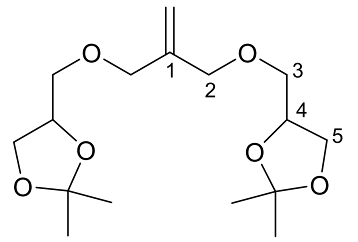

G[1.1]-ene was synthesized as described after general procedure A with solketal nr (7.97 g, 60.3 mmol) and methallyl dichloride nr (3.94 g, 30.9 mmol) in 200 mL dry tetrahydrofuran. Column chromatography was performed with cyclohexane/ethyl acetate 4:1. The product was isolated as a colorless oil.

Yield: 8.85 g (28.0 mmol, 91 %), Ref. (*44*): 99 %

FT-IR (ATR): $\tilde{\nu}$ = 2987 (m), 2871 (m), 1736 (w), 1456 (w), 1371 (s), 1215 (s), 1085 (vs), 1051 (vs), 975 (m), 919 (m), 842 (s), 790 (w), 751 (w), 514 (m) cm^{-1}.

^{1}H-NMR: (600 MHz, $CDCl_3$, 298 K): δ = 5.19 (s, 2 H, CH_2), 4.27 (quint., ^3J = 6.0 Hz, 2 H, *H*-4), 4.07-4.02 (m, 6 H, *H*-2, *H*-5), 3.75-3.72 (m, 2 H, *H*-5), 3.57-3.48 (m, 4 H, *H*-3), 1.42 (s, 6 H, CH_3), 1.36 (s, 6 H, CH_3) ppm.

^{13}C-NMR: (150 MHz, $CDCl_3$, 298 K): δ = 142.1 (*C*-1), 114.8 (*C*H_2), 109.6 (*C*$(CH_3)_2$), 74.8 (*C*-4), 72.2 (*C*-2), 71.3 (*C*-3), 66.9 (*C*-5), 26.9 (*C*H_3), 25.5 (*C*H_3) ppm.

MS (EI-ToF): *m/z* (%) = 301.19 (20) $[M-CH_3]^+$.

G[1.1]-OH

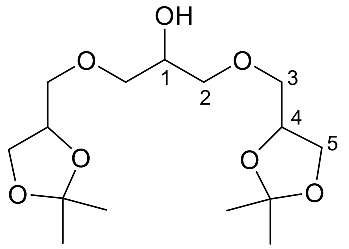

G[1.1]-OH was synthesized as described after general procedure B with G[1.1]-ene (8.50 g, 26.9 mmol) in 200 mL dry methanol/dichloromethane 1:1. The product was isolated as a colorless oil.
Yield: 8.23 g (25.7 mmol, 95 %), Ref. (*44*): 99 %
FT-IR (ATR): $\tilde{\nu}$ = 3475 (br. w), 2986 (m), 2875 (m), 1715 (w), 1456 (w), 1371 (s), 1252 (m), 1212 (s), 1076 (vs), 1049 (vs), 975 (m), 841 (s), 792 (w), 514 (s) cm^{-1}.
^{1}H-NMR: (500 MHz, $CDCl_3$, 298 K): δ = 4.31-4.23 (m, 2 H, *H*-4), 4.07-4.03 (m, 2 H, *H*-5), 3.99-3.95 (m, 1 H, *H*-1), 3.74-3.70 (m, 2 H, *H*-5), 3.59-3.51 (m, 8 H, *H*-3, *H*-2), 1.42 (s, 6 H, CH_3), 1.36 (s, 6 H, CH_3) ppm.
^{13}C-NMR: (125 MHz, $CDCl_3$, 298 K): δ = 109.6 ($C(CH_3)_2$), 74.8 (*C*-4), 72.9 (*C*-2), 72.6 (*C*-3), 69.6 (*C*-1), 66.7 (*C*-5), 26.9 (CH_3), 25.5 (CH_3) ppm.
HR-EI-MS: *m/z* $[M+H]^+$ berechnet für $C_{15}H_{29}O_7$: 321.19133; gefunden: 321.19126 (-0.19 ppm).

G[2.1]-ene

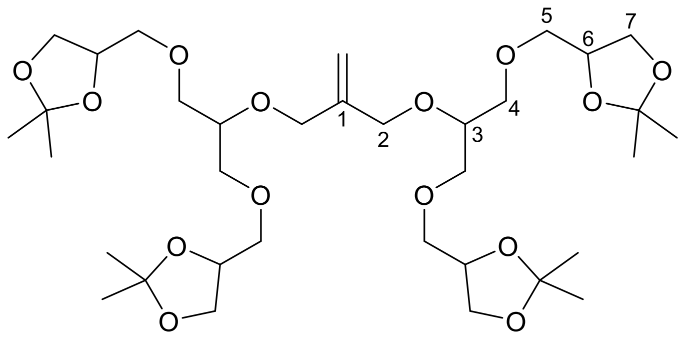

G[1.1]-ene was synthesized as described after general procedure A with G[1.1]-OH (8.08 g, 25.2 mmol) and methallyl dichloride nr (1.57 g, 12.5 mmol) in 100 mL dry tetrahydrofuran. Column chromatography was performed with cyclohexane/ethyl acetate 1:2. The product was isolated as a colorless oil.
Yield: 7.64 g (11.0 mmol, 88 %), Ref. (*44*): 94 %
FT-IR (ATR): $\tilde{\nu}$ = 2986 (m), 2874 (m), 1736 (w), 1457 (w), 1370 (s), 1251 (m), 1213 (s), 1078 (vs), 1051 (vs), 975 (m), 917 (m), 843 (s), 792 (w), 514 (m) cm^{-1}.
^{1}H-NMR: (200 MHz, Acetone-d_6, 298 K): δ = δ 5.16 (quint., ^{4}J = 1.2 Hz, 2 H, CH_2), 4.31-4.11 (m, 8 H, *H*-2, *H*-6), 4.09-3.97 (m, 4 H, *H*-7), 3.76-3.66 (m, 4 H, *H*-5), 3.66-3.41 (m, 18 H, *H*-3, *H*-4, *H*-5, *H*-7), 1.36-1.31 (m, 12 H, CH_3), 1.30-1.26 (s, 12 H, CH_3) ppm.
HR-EI-MS: *m/z* $[M+Na]^+$ berechnet für $C_{34}H_{60}O_{14}Na$: 715.38753; gefunden: 715.38584 (-2.36 ppm).

G[2.1]-OH

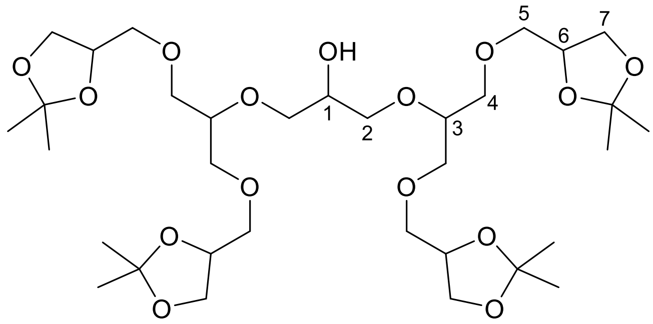

G[1.1]-OH was synthesized as described after general procedure B with G[1.1]-ene (7.64 g, 11.0 mmol) in 100 mL dry methanol/dichloromethane 1:1. The product was isolated as a colorless oil.

Yield: 7.32 g (10.5 mmol, 96 %), Ref. (*44*): 99.9 %

FT-IR (ATR): $\tilde{\nu}$ = 3476 (br. w), 2985 (m), 2921 (s), 2856 (m), 1736 (w), 1457 (m), 1371 (s), 1213 (s), 1051 (vs), 975 (m), 841 (s), 792 (w), 750 (m), 514 (m) cm^{-1}.

^{1}H-NMR: (500 MHz, Aceton-d_6, 298 K): δ = 4.25-4.17 (m, 4 H, *H*-6), 4.05-4.00 (m, 4 H, *H*-7), 3.81-3.77 (m, 1 H, *H*-1), 3.73-3.69 (m, 4 H, *H*-5), 3.67-3.63 (m, 4 H, *H*-4), 3.61-3.47 (m, 18 H, *H*-2, *H*-3, *H*-4, *H*-5, *H*-7), 1.34 (s, 12 H, CH_3), 1.28 (s, 12 H, CH_3) ppm.

^{13}C-NMR: (125 MHz, Aceton-d_6, 298 K): δ = 109.6 (*C*(CH_3), 79.5 (*C*-4), 75.6 (*C*-6), 73.2 (*C*-2), 72.8 (*C*-5), 72.3 (*C*-3) 70.7 (*C*-1), 67.4 (*C*-7), 27.2 (CH_3), 25.8 (CH_3) ppm.

HR-EI-MS: *m/z* $[M+H]^+$ berechnet für $C_{33}H_{61}O_{15}$: 697.40050; gefunden: 697.39982 (-0.97 ppm).

G[2.0]-OH

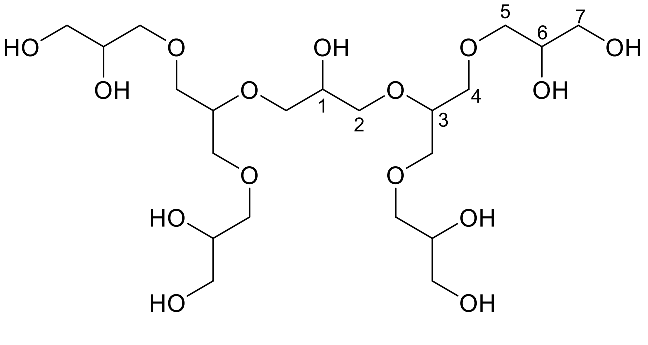

G[2.1]-OH (756 mg, 1.08 mmol) was dissolved in a 50 ml mixture of acetic acid/H_2O/MeOH (2:2:1). After stirring at room temperature, the solvent was removed under reduced pressure. The product was obtained as a viscous oil.

Yield: 574 mg (1.07 mmol, 99 %)

^{1}H-NMR: (500 MHz, D_2O, 298 K): δ = 4.01-3.96 (m, 1 H, *H*-1), 3.92-3-85 (m, 4 H, *H*-6), 3.82-3.77 (m, 2 H, *H*-3), 3.76-3.67 (m, 6 H, *H*-2, *H*-4), 3.67-3.59 (m, 14 H, *H*-2, *H*-4, *H*-5, *H*-7), 3.59-3.50 (m, 8 H, *H*-5, *H*-7) ppm.

^{13}C-NMR: (125 MHz, D_2O, 298 K): δ = 78.5 (*C*-3), 72.7 (*C*-7), 71.5 (*C*-2), 71.0 (*C*-4), 70.9 (*C*-6), 70.0 (*C*-1), 63.2 (*C*-5) ppm.

Sample for hyperpolarization: We dissolved 261.86 mg (122 mM) of G[2.0]-OH type dendrons (MW 536.57 g/mol) in 4 mL water to yield a concentration of 128 mM and diluted the dendrons for the other experiments by 8 (15.3 mM), 16 (7.6 mM) and 48 (2.5 mM) times.

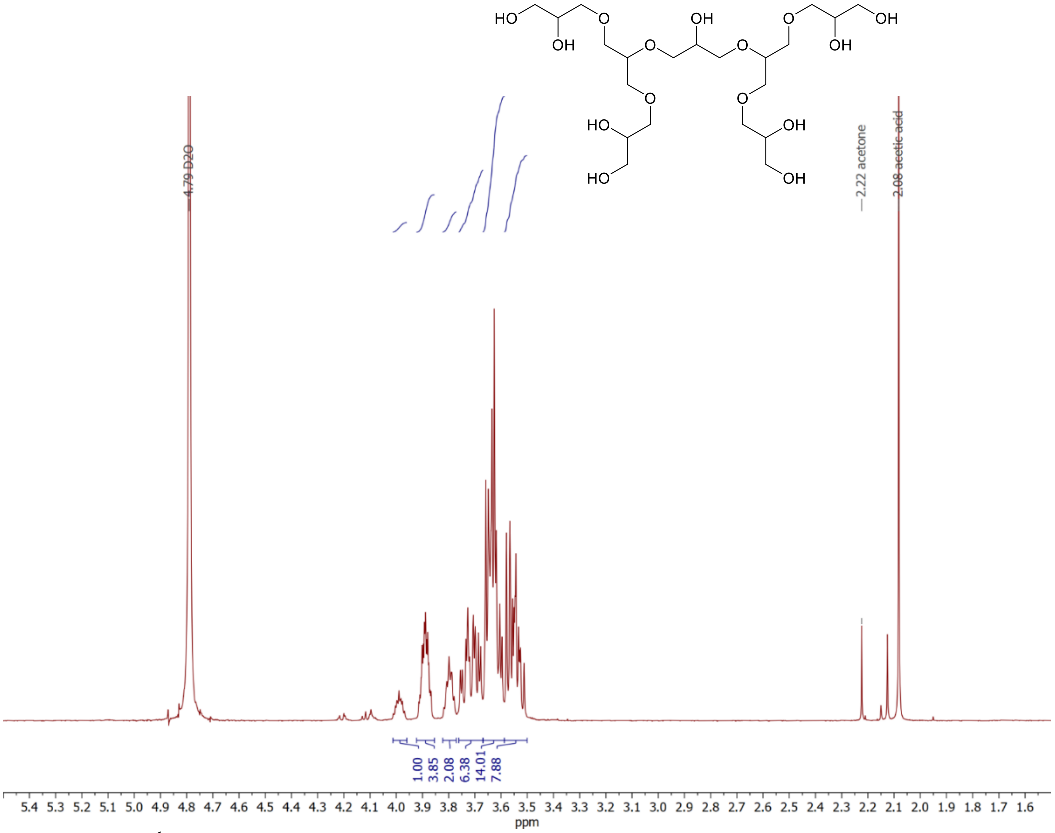

Figure S9: ^{1}H-NMR of G[2.0]-OH.

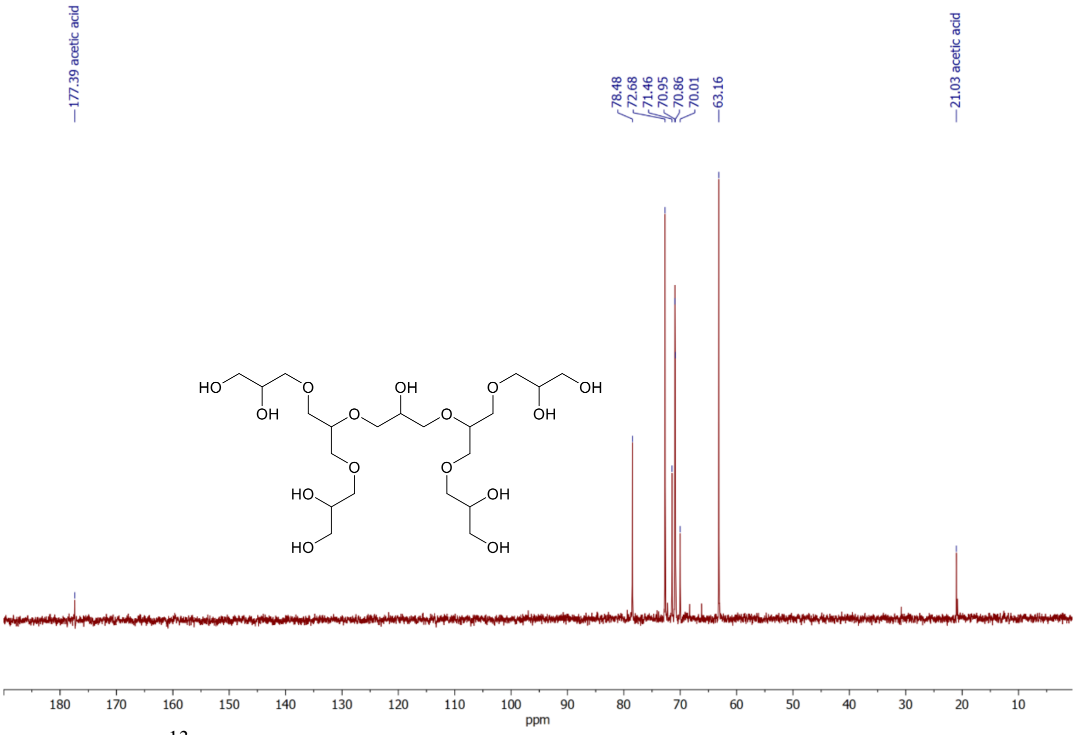

Figure S10. ^{13}C-NMR of G[2.0]-OH.

S9. <u>Spectra of ^{15}N tracers</u>

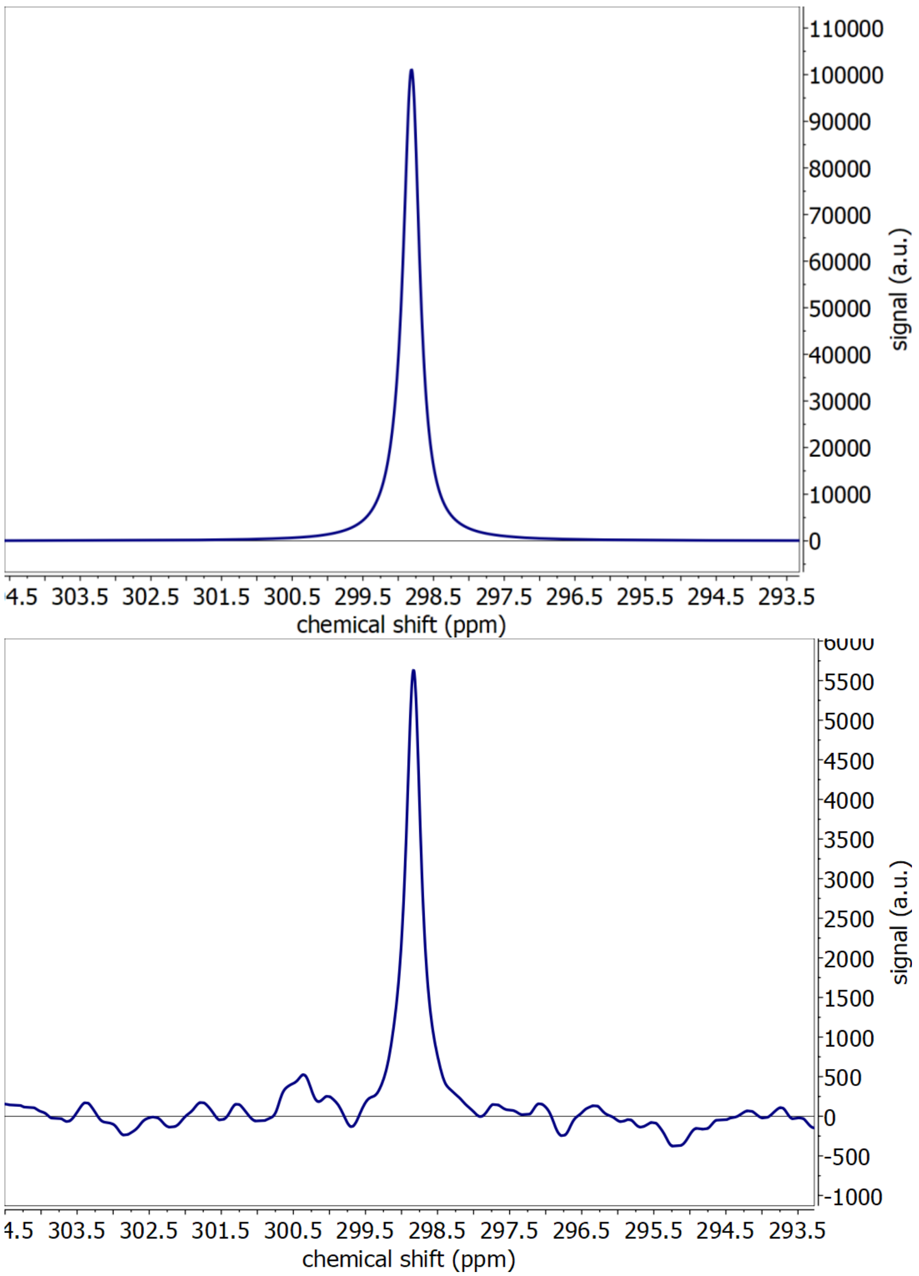

Figure S11: Spectrum of hyperpolarized to 24.6% 1-^{15}N-nicotinamide, acquired with a 5° flip angle and receiver gain 10 (top) and corresponding thermal spectrum measured using 64 averages as described in methods (bottom). Experiment 173, **Tab. S4**.

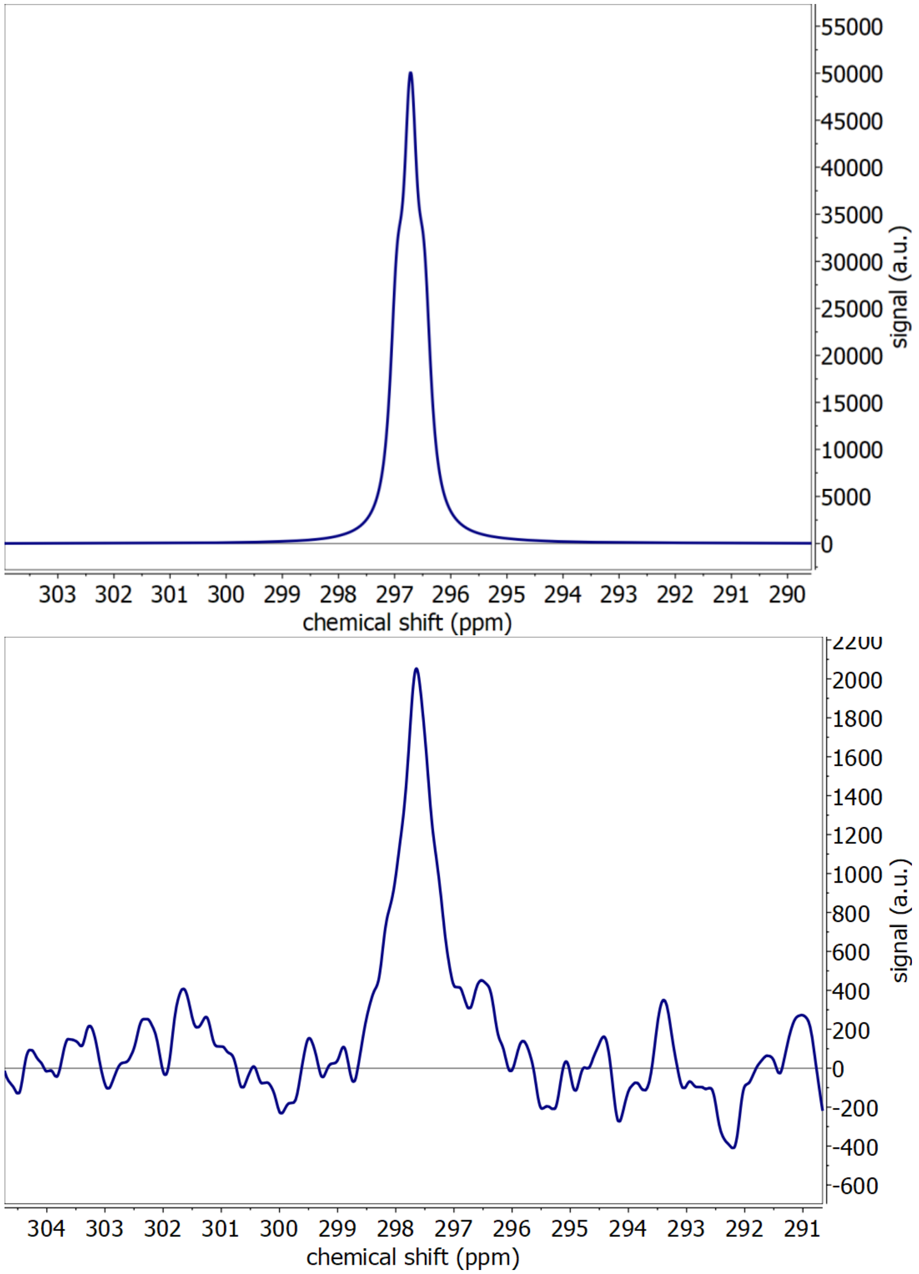

Figure S12: Spectrum of hyperpolarized to 21.1% ^{15}N-pyridine, acquired with a 5° flip angle and receiver gain of 10 (top) and corresponding thermal spectrum measured using 64 averages as described in methods (bottom). Experiment 2, **Tab. S8**.

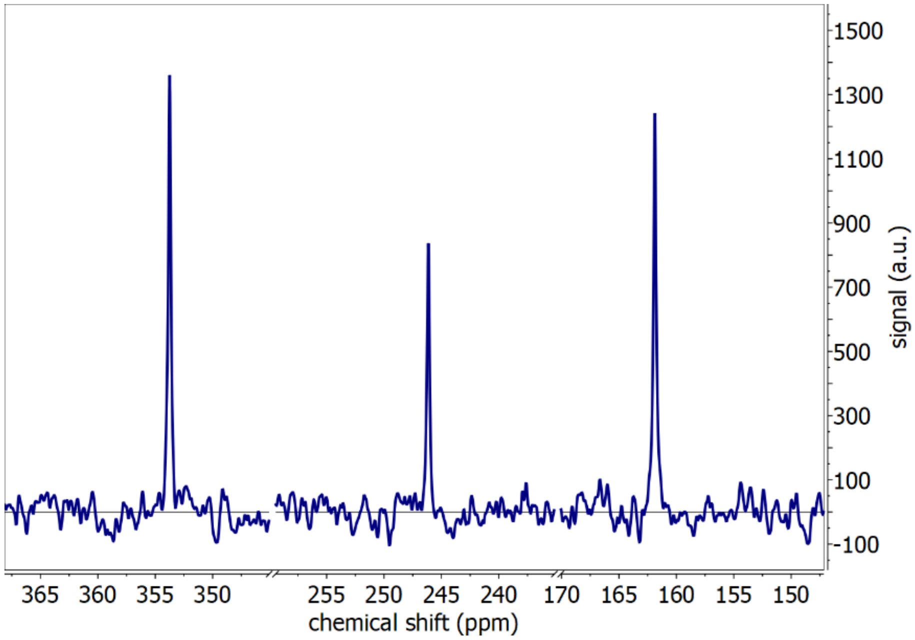

Figure S13: Spectrum of hyperpolarized naturally abundant metronidazole, acquired with a 5° flip angle and receiver gain of 101, thermal spectra were not measured in this case. Experiment 3, **Tab. S9 and S10**.

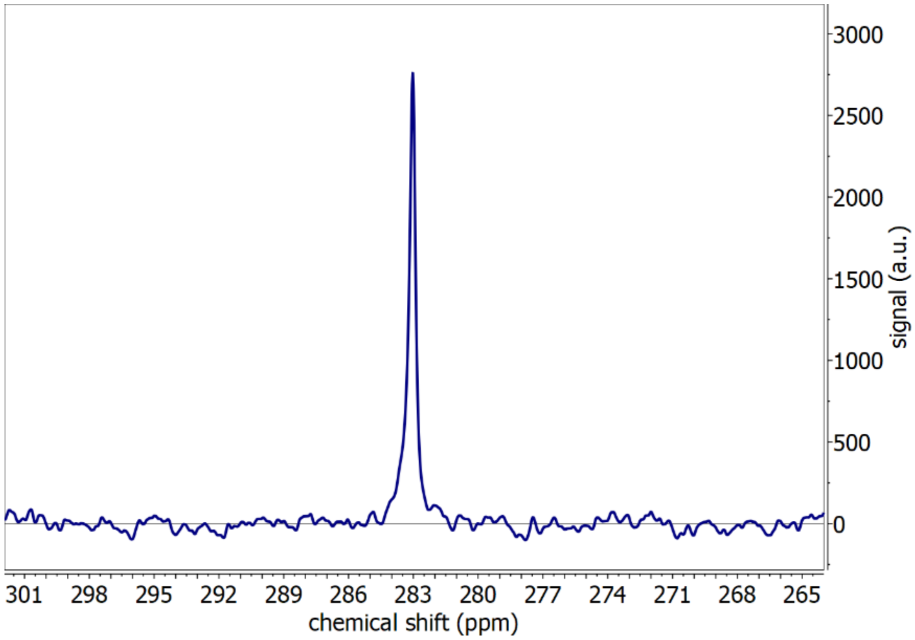

Figure S14: Spectrum of hyperpolarized naturally abundant pyrimidine, acquired with a 5° flip angle and receiver gain of 101, thermal spectra were not measured in this case. Experiment 3, **Tab. S11**.

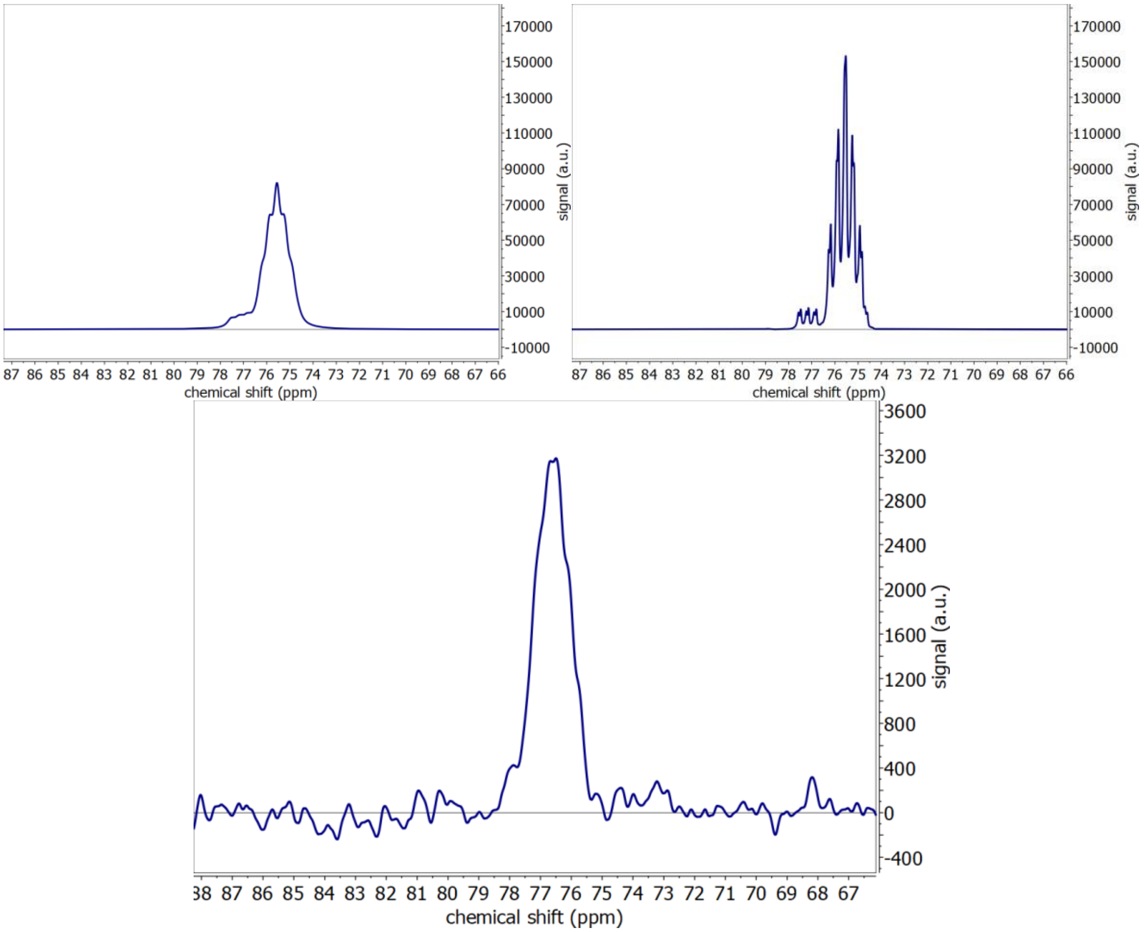

Figure S15: Spectrum of hyperpolarized to 13.7% $^{15}N_2$-urea, acquired with a 5° flip angle and receiver gain of 10 (top, with line broadening of 10 Hz on the left and 0 Hz on the right). The corresponding thermal spectrum (bottom) was measured using 64 signal averages under the same conditions as described in the text and presented with the same line broadening parameters, respectively. Both spectra were obtained in D_2O (experiment 15, **Tab. S12)**. The observed additional doublet-of-triplet pattern on the left side arises from partial protonation of the solvent, due to the addition of protons in urea, H_2O, trehalose, and radical in the DNP sample before dissolution and non-deuterated buffer in the dissolution medium.

S10. Data on the main text figures

The duration of the manual transfer, t, from the detection site was recorded for each experiment and found to vary by a few seconds. To reduce the effect of these variations on the polarization, we calculated the mean transfer time, t_{avg}, for each series. Then, we assumed a uniform T_1 relaxation during transfer, and extrapolated the polarization of each experiment to this (average) time:

$$P_{\mathrm{corrected}}(t \rightarrow t_{\mathrm{avg}}) = P(t) \cdot \exp\left(\frac{t - t_{\mathrm{avg}}}{T_1}\right).$$

When, for example, the sample experienced a longer relaxation time compared to the average, $t > t_{\mathrm{avg}}$, then the correction factor is greater than 1 to compensate for this. In the opposite case, the correction factor would be smaller. Of course, T_1 depends on the magnetic field, $T_1(B_0)$, which is generally not known for the sample during the transfer. We approximated it by using T_1(0.57T) for Tables S3 to S7 (Fig. 2, main text). For Tables S8 to S14 (Fig. 3 main text), the T_1 at 9.4 T was used – because several molecules were at natural abundance, the 0.57 T MRI was not sensitive enough to provide a low field T1 reliably.

Furthermore, in the case of naturally abundant tracers (Tables S10 to S12), no thermal signal could be acquired with sufficient SNR; hence, the polarization could not be calculated. Instead, the absolute signal amplitude of the first hyperpolarized signal was taken, corrected using the equation above, and then normalized to the highest signal in the table.

Table S3: Figure 2B with NAM as CIDER agent and 1-^{15}N-NAM as a tracer. The average transfer time was 18.1 s.

[1-14N-NAM] (M)	Experiment	pH	transfer time (s)	polarization detected (%)	polarization corrected (%)	T1 N1 0.57 T (s)	T1 N1 1 T (s)	T1 N1 9.4 T (s)	T1 Calpha 1 T (s)	T1 C4 1 T (s)
0	158	8.86	17.3	0.92	0.89	25.8	38.8	25.0	33.8	38.4
0.125	157	8.81	17.8	0.97	0.95	17.3	21.4	19.4	32.5	37.3
0.25	155	8.82	17.0	0.86	0.79	12.7	15.1	14.4	31.1	35.4
0.5	154	8.59	18.5	3.82	3.89	22.1	26.3	17.7	29.2	39.4
1	152	8.62	17.0	6.68	6.37	22.9	27.4	15.5	28.5	39.3
2	156	8.63	21.0	2.50	2.95	17.4	19.0	8.6	17.5	25.8

Table S4: Figure 2C with urea as CIDER agent and 1-^{15}N-NAM as a tracer. The average transfer time was 18.9 s.

[Urea] (M)	Experiment	pH	transfer time (s)	polarization detected (%)	polarization corrected (%)	T1 N1 0.57 T (s)	T1 N1 1 T (s)	T1 N1 9.4 T (s)	T1 Calpha 1 T (s)	T1 C4 1 T (s)
0	175	9.42	19.0	5.11	5.13	44.5	93.6	31.0	39.3	45.4
0.5	174	9.19	19.0	28.56	28.61	100.2	127.6	35.6	44.6	46.5
1	173	9.51	19.0	24.56	24.60	98.0	133.6	34.1	34.1	41.8
2	172	9.21	19.0	21.19	21.22	105.5	128.0	30.7	34.1	42.8
4	168	9.48	18.0	18.68	18.43	61.7	77.8	25.5	31.3	39.8
8	171	9.50	19.0	12.82	12.86	58.7	74.0	17.9	17.1	27.8

Table S5: Figure 2D with glycerol as CIDER agent and 1-^{15}N-NAM as a tracer. The average transfer time was 16.6 s.

[Glycerol] (M)	Experiment	pH	transfer time (s)	polarization detected (%)	polarization corrected (%)	T1 N1 0.57 T (s)	T1 N1 1 T (s)	T1 N1 9.4 T (s)	T1 Calpha 1 T (s)	T1 C4 1 T (s)
0	187	7.57	16.0	0.000	0.000	no signal	no signal	no signal	39.4	40.9
0.05	190	7.55	17.1	0.183	0.216	no signal	no signal	17.0	41.1	46.6
0.1	191	7.43	16.6	0.730	0.733	8.1	9.9	21.0	no exp	no exp
0.25	192	7.56	16.3	1.915	1.854	9.6	11.2	23.0	43.1	50.2
0.5	193	7.52	15.8	2.018	1.861	10.0	11.0	21.5	41.7	48.2
1	194	7.41	16.8	1.493	1.522	9.9	12.3	19.5	36.7	42.7
0	195	7.54	17.9	3.426	3.685	18.4	22.1	27.6	43.0	51.0
0	196	7.48	16.8	2.491	2.524	18.4	21.9	26.4	44.0	53.5
0	197	7.44	15.9	0.015	0.012	no signal	no signal	20.2	42.3	47.5

Table S6: Figure 2D with glycerol as CIDER agent and 1-^{15}N-NAM as a tracer. Comparison of solid-state ^{15}N signal and liquid state polarization.

Experiment	solid-state signal (a.u.)	liquid-state polarization (%)
187	31,675	0.000
190	33,600	0.216
191	32,850	0.733
192	27,770	1.854
193	28,500	1.861
194	26,560	1.522
195	26,313	3.685
196	26,044	2.524
197	24,858	0.012

Table S7: Figure 2E with dendrons as CIDER agent and 1-^{15}N-NAM as tracer. The average transfer time was 15.7 s.

[Dendrons] (M)	Experiment	pH	transfer time (s)	polarization detected (%)	polarization corrected (%)	T1 N1 0.57 T (s)	T1 N1 1 T (s)	T1 N1 9.4 T (s)	T1 Calpha 1 T (s)	T1 C4 1 T (s)
0	197	7.44	15.9	0.015	0.016	no signal	no signal	20.2	42.3	47.5
2.54	202	7.54	16.1	3.064	3.120	21.1	26.8	26.9	39.8	47.3
7.63	201	7.61	15.0	1.075	1.026	15.2	16.4	23.8	43.3	52.3
15.25	200	7.58	15.6	0.049	0.047	no signal	no signal	18.1	42.5	50.0
122.01	198	7.66	16.0	0.000	0.000	no signal	no signal	no signal	34.1	30.8

Table S8: Figure 3A with 1-^{15}N-NAM as a tracer. The average transfer time was 18.6 s.

Dissolution medium	Experiment	pH	transfer time (s)	polarization detected (%)	polarization corrected (%)	T1 N1 0.57 T (s)	T1 N1 1 T (s)	T1 N1 9.4 T (s)	T1 Calpha 1 T (s)	T1 C4 1 T (s)
H2O	175	9.42	19.0	5.11	5.18	44.5	93.6	31.0	39.3	45.4
H2O + 1 M Urea	173	9.51	19.0	24.56	24.85	98.0	133.6	34.1	34.1	41.8
H2O	159	7.52	18.7	0.02	0.02	no signal	no signal	16.4	36.6	39.1
D2O	65	7.45	none	0.00	0.00	no signal	no signal	no signal	no exp	no exp
H2O + 1 M NAM	160	7.50	17.7	0.29	0.27	5.8	7.0	11.3	27.1	33.5
D2O + 1 M NAM	no exp	no exp	no exp	no exp	no exp	no exp	no exp	no exp	no exp	no exp

Table S9: Figure 3B with ^{15}N-pyridine as a tracer. The average transfer time was 17.1 s.

Dissolution medium	Experiment	pH	transfer time (s)	polarization detected (%)	polarization corrected (%)	T1 N 0.57 T (s)	T1 N 1 T (s)	T1 N 9.4 T (s)
H2O	1	9.39	17.0	7.83	7.81	20.0	63.8	56.0
H2O + 1 M Urea	2	9.38	16.8	21.14	21.05	68.9	122.0	72.4
H2O	5	7.53	18.0	0.03	0.04	no signal	no signal	15.9
D2O	6	7.54	17.0	4.63	4.62	7.6	12.8	38.7
H2O + 1 M NAM	4	7.53	16.9	2.46	2.44	6.3	8.7	24.3
D2O + 1 M NAM	7	7.52	17.0	8.11	8.08	10.6	15.6	29.2

Table S10: Figure 3C with naturally abundant MNZ (3-N) as a tracer. The average transfer time was 17.3 s.

Dissolution medium	Experiment	pH	transfer time (s)	signal detected (a.u.)	signal normalized (a.u.)	T1 N3 0.57 T (s)	T1 N3 1 T (s)	T1 N3 9.4 T (s)
H2O	3	9.42	18.0	0.004316	1.00	249.2	119.8	55.1
H2O + 1 M Urea	7	9.37	17.3	0.001872	0.43	143.2	230.0	49.2
H2O	4	7.50	16.7	0.000000	0.00	no signal	no signal	38.2
D2O	8	7.83	17.9	0.000000	0.00	no signal	71.7	45.4
H2O + 1 M NAM	5	7.44	17.0	0.002084	0.47	no signal	91.1	41.6
D2O + 1 M NAM	10	7.78	17.1	0.002927	0.66	no signal	48.3	27.6

Table S11: Figure 3C with naturally abundant MNZ (NO_2) as a tracer. The average transfer time was 17.3 s.

Dissolution medium	Experiment	pH	transfer time (s)	signal detected (a.u.)	signal normalized (a.u.)	T1 NO2 0.57 T (s)	T1 NO2 1 T (s)	T1 NO2 9.4 T (s)
H2O	3	9.42	18.0	0.00560	0.9857	386.4	476.7	96.7
H2O + 1 M Urea	7	9.37	17.3	0.00352	0.6151	346.2	397.8	98.6
H2O	4	7.50	16.7	0.00477	0.8291	213.3	457.5	111.6
D2O	8	7.83	17.9	0.00569	1.0000	no signal	484.1	105.6
H2O + 1 M NAM	5	7.44	17.0	0.00454	0.7893	183.0	301.1	60.7
D2O + 1 M NAM	10	7.78	17.1	0.00523	0.9094	no signal	326.7	43.6

Table S12: Figure 3D with naturally abundant pyrimidine as a tracer. The average transfer time was 17.3 s.

Dissolution medium	Experiment	pH	transfer time (s)	signal detected (a.u.)	signal normalized (a.u.)	T1 N 0.57 T (s)	T1 N 1 T (s)	T1 N 9.4 T (s)
H2O	2	9.42	16.2	0.005446	0.452	83.3	158.7	80.8
H2O + 1 M Urea	3	9.28	19.0	0.011651	1.000	234.1	214.3	82.5
H2O	4	7.58	16.0	0.005061	0.417	31.7	23.9	65.8
D2O	5	7.73	17.0	0.007990	0.669	59.7	65.3	76.8
H2O + 1 M NAM	7	7.48	18.0	0.010789	0.922	73.0	83.4	43.9
D2O + 1 M NAM	6	7.49	17.4	0.009698	0.818	73.0	63.8	39.0

Table S13: Figure 3E with $^{15}N_2$-urea as tracer. The average transfer time was 16.7 s.

Dissolution medium	Experiment	pH	transfer time (s)	polarization detected (%)	polarization corrected (%)	T1 N 0.57 T (s)	T1 N 1 T (s)	T1 N 9.4 T (s)
H2O	9	9.5	17.0	9.67	9.77	27.72	36.11	29.54
H2O + 1 M Urea	10	9.4	17.0	8.72	8.82	27.56	29.88	27.36
H2O	13	7.4	15.0	8.79	8.31		32.22	30.59
D2O	15	7.7	17.0	13.73	13.75	175.56	271.76	178.68
H2O + 1 M NAM	14	7.4	17.5	8.21	8.49	22.17	23.55	23.9
D2O + 1 M NAM	no exp	no exp	no exp	no exp	no exp	no exp	no exp	no exp

S11. Other combinations of hyperpolarized molecules with potential CIDER agents

Case 1. We used 2,2'-bipyridine as a CIDER agent with NAM. No significant preservation of polarization was observed. This negative result is likely due to the low solubility of 2,2'-bipyridine in water: 5.5 g/L ~ 35 mM and that it is not a hydrogen bond donor, although 2,2'-bipyridine is an excellent chelating agent with two coordination sites.

Case 2. Ammonia has some loose similarities with urea and very high solubility. However, even at concentrations of 1 M (dissolution medium buffered to pH 7.4, pH 6.8 after dissolution), no significant effect on the preservation of ^{15}N-pyridine polarization was found. The interaction with pyridine is likely too weak because amide hydrogens of urea or NAM are much better hydrogen donors than protonated ammonia in the case of neutral pH (ammonia pKa 9.24). Since pH values above the pKa of ammonia are not physiological, ammonia does not present a viable CIDER agent in our case. Although varying concentrations of ammonia are produced during the heating of urea inside the dissolution media, the above findings suggest that mostly urea participates in the CIDER effect of the formed solution.

Case 3. Jiang et al. reported T_1 for NAM at pH 5.9 of around 22 s, though also with low polarization of about 1.2. Previously, we could not reproduce the hyperpolarized 1-^{15}N-NAM signal at this pH as no signal was observed (20). However, we suspect now that the difference in the sample composition can be the reason: Jiang et al. had about 145 mM concentration of DMSO, which can act as a CIDER agent due to its very strong dipole moment, possibly explaining the difference in findings: instead of DMSO, we used water as a solvent.

Case 4. We used 1 M NAM and urea as CIDER agents trying to preserve the hyperpolarization of 1-^{13}C-pyruvate and glucose-d7 – no significant positive effects on the preservation of polarization were found. More experiments are needed to deduct if the CIDER agents make a difference or not. Possibly, the concentration of the CIDER agents was too high or not appropriate for these compounds: similar detrimental effects at high concentrations were discussed for 1-^{15}N-NAM (**Fig. 2** main text). Studies of other agents' effect on pyruvate relaxation are ongoing.

REFERENCES AND NOTES

1. S. J. Nelson, J. Kurhanewicz, D. B. Vigneron, P. E. Z. Larson, A. L. Harzstark, M. Ferrone, M. van Criekinge, J. W. Chang, R. Bok, I. Park, G. Reed, L. Carvajal, E. J. Small, P. Munster, V. K. Weinberg, J. H. Ardenkjaer-Larsen, A. P. Chen, R. E. Hurd, L.-I. Odegardstuen, F. J. Robb, J. Tropp, J. A. Murray, Metabolic imaging of patients with prostate cancer using hyperpolarized [1-^{13}C]pyruvate. *Sci. Transl. Med.* **5**, 198ra108 (2013).

2. C. H. Cunningham, J. Y. C. Lau, A. P. Chen, B. J. Geraghty, W. J. Perks, I. Roifman, G. A. Wright, K. A. Connelly, Hyperpolarized ^{13}C metabolic MRI of the human heart: Initial experience. *Circ. Res.* **119**, 1177–1182 (2016).

3. R. Woitek, K. M. Brindle, Hyperpolarized carbon-13 MRI in breast cancer. *Diagnostics* **13**, 2311 (2023).

4. J. H. Ardenkjær-Larsen, S. Bowen, J. R. Petersen, O. Rybalko, M. S. Vinding, M. Ullisch, N. C. Nielsen, Cryogen-free dissolution dynamic nuclear polarization polarizer operating at 3.35 T, 6.70 T, and 10.1 T. *Magn. Reson. Med.* **81**, 2184–2194 (2019).

5. E. Cavallari, C. Carrera, M. Sorge, G. Bonne, A. Muchir, S. Aime, F. Reineri, The ^{13}C hyperpolarized pyruvate generated by ParaHydrogen detects the response of the heart to altered metabolism in real time. *Sci. Rep.* **8**, 8366 (2018).

6. T. L. K. Hune, S. Mamone, A. B. Schmidt, I. Mahú, N. D'Apolito, D. Wiedermann, J. Brüning, S. Glöggler, Hyperpolarized multi-organ spectroscopy of liver and brain using 1-^{13}C-pyruvate enhanced via parahydrogen. *Appl. Magn. Reson.* **54**, 1283–1295 (2023).

7. K. MacCulloch, A. Browning, D. O. Guarin Bedoya, S. J. McBride, M. B. Abdulmojeed, C. Dedesma, B. M. Goodson, M. S. Rosen, E. Y. Chekmenev, Y.-F. Yen, P. TomHon, T. Theis, Facile hyperpolarization chemistry for molecular imaging and metabolic tracking of [1-^{13}C] pyruvate in vivo. *J. Magn. Reson. Open* **16-17**, 100129 (2023).

8. H. de Maissin, P. R. Groß, O. Mohiuddin, M. Weigt, L. Nagel, M. Herzog, Z. Wang, R. Willing, W. Reichardt, M. Pichotka, L. Heß, T. Reinheckel, H. J. Jessen, R. Zeiser, M. Bock, D. von Elverfeldt, M. Zaitsev, S. Korchak, S. Glöggler, J.-B. Hövener, E. Y. Chekmenev, F.

Schilling, S. Knecht, A. B. Schmidt, In vivo metabolic imaging of [1-^{13}C]pyruvate-d3 hyperpolarized by reversible exchange with parahydrogen. *Angew. Chem. Int. Ed. Engl.* **62**, e202306654 (2023).

9. C. Hundshammer, M. Grashei, A. Greiner, S. J. Glaser, F. Schilling, pH dependence of T_1 for ^{13}C-labelled small molecules commonly used for hyperpolarized magnetic resonance imaging. *ChemPhysChem* **20**, 798–802 (2019).

10. A. Ferrari, J. Peters, M. Anikeeva, A. N. Pravdivtsev, F. Ellermann, K. Them, O. Will, E. Peschke, H. Yoshihara, O. Jansen, J.-B. Hövener, Performance and reproducibility of ^{13}C and ^{15}N hyperpolarization using a cryogen-free DNP polarizer. *Sci. Rep.* **12**, 11694 (2022).

11. J. Singh, E. H. Suh, G. Sharma, C. Khemtong, A. D. Sherry, Z. Kovacs, Probing carbohydrate metabolism using hyperpolarized ^{13}C-labeled molecules. *NMR Biomed.* **32**, e4018 (2018).

12. T. B. Rodrigues, E. M. Serrao, B. W. C. Kennedy, D.-E. Hu, M. I. Kettunen, K. M. Brindle, Magnetic resonance imaging of tumor glycolysis using hyperpolarized ^{13}C-labeled glucose. *Nat. Med.* **20**, 93–97 (2014).

13. C. von Morze, J. A. Engelbach, G. D. Reed, A. P. Chen, J. D. Quirk, T. Blazey, R. Mahar, C. R. Malloy, J. R. Garbow, M. E. Merritt, ^{15}N-carnitine, a novel endogenous hyperpolarized MRI probe with long signal lifetime. *Magn. Reson. Med.* **85**, 1814–1820 (2021).

14. H.-K. Kim, G. H. Lee, Y. Chang, Gadolinium as an MRI contrast agent. *Future Med. Chem.* **10**, 639–661 (2018).

15. M. Carravetta, O. G. Johannessen, M. H. Levitt, Beyond the T_1 limit: Singlet nuclear spin states in low magnetic fields. *Phys. Rev. Lett.* **92**, 153003 (2004).

16. M. C. D. Tayler, M. H. Levitt, Paramagnetic relaxation of nuclear singlet states. *Phys. Chem. Chem. Phys.* **13**, 9128–9130 (2011).

17. A. K. Grant, E. Vinogradov, Long-lived states in solution NMR: Theoretical examples in three- and four-spin systems. *J. Magn. Reson.* **193**, 177–190 (2008).

18. M. H. Levitt, Singlet nuclear magnetic resonance. *Annu. Rev. Phys. Chem.* **63**, 89–105 (2012).

19. G. Stevanato, J. T. Hill-Cousins, P. Håkansson, S. S. Roy, L. J. Brown, R. C. D. Brown, G. Pileio, M. H. Levitt, A nuclear singlet lifetime of more than one hour in room-temperature solution. *Angew. Chem. Int. Ed. Engl.* **54**, 3740–3743 (2015).

20. A. N. Pravdivtsev, A. V. Yurkovskaya, H. Zimmermann, H.-M. Vieth, K. L. Ivanov, Magnetic field dependent long-lived spin states in amino acids and dipeptides. *Phys. Chem. Chem. Phys.* **16**, 7584–7594 (2014).

21. S. Korchak, S. Mamone, S. Glöggler, Over 50 % ^{1}H and ^{13}C polarization for generating hyperpolarized metabolites–A *para*-hydrogen approach. *ChemistryOpen* **7**, 672–676 (2018).

22. A. Brahms, A. N. Pravdivtsev, T. Stamp, F. Ellermann, F. Sönnichsen, J.-B. Hövener, R. Herges, Synthesis of ^{13}C and ^{2}H labeled vinyl pyruvate and hyperpolarization of pyruvate. *Chem. A Eur. J.* **28**, e202201210 (2022).

23. J. R. Birchall, M. S. H. Kabir, O. G. Salnikov, N. V. Chukanov, A. Svyatova, K. V. Kovtunov, I. V. Koptyug, J. G. Gelovani, B. M. Goodson, W. Pham, E. Y. Chekmenev, Quantifying the effects of quadrupolar sinks via ^{15}N relaxation dynamics in metronidazoles hyperpolarized via SABRE-SHEATH. *Chem. Commun.* **56**, 9098–9101 (2020).

24. P. Miéville, P. Ahuja, R. Sarkar, S. Jannin, P. R. Vasos, S. Gerber-Lemaire, M. Mishkovsky, A. Comment, R. Gruetter, O. Ouari, P. Tordo, G. Bodenhausen, Scavenging free radicals to preserve enhancement and extend relaxation times in NMR using dynamic nuclear polarization. *Angew. Chem. Int. Ed. Engl.* **49**, 6182–6185 (2010).

25. T. El Daraï, S. Jannin, Sample formulations for dissolution dynamic nuclear polarization. *Chem. Phys. Rev.* **2**, 041308 (2021).

26. M. Negroni, E. Turhan, T. Kress, M. Ceillier, S. Jannin, D. Kurzbach, Frémy's salt as a low-persistence hyperpolarization agent: Efficient dynamic nuclear polarization plus rapid radical scavenging. *J. Am. Chem. Soc.* **144**, 20680–20686 (2022).

27. A. Capozzi, M. Karlsson, J. R. Petersen, M. H. Lerche, J. H. Ardenkjaer-Larsen, Liquid-state ^{13}C polarization of 30% through photoinduced nonpersistent radicals. *J. Phys. Chem. C* **122**, 7432–7443 (2018).

28. R. M. Malinowski, K. W. Lipsø, M. H. Lerche, J. H. Ardenkjær-Larsen, Dissolution dynamic nuclear polarization capability study with fluid path. *J. Magn. Reson.* **272**, 141–146 (2016).

29. P. M. Wax, Current use of chelation in american health care. *J. Med. Toxicol.* **9**, 303–307 (2013).

30. J.-B. Hövener, E. Y. Chekmenev, K. C. Harris, W. H. Perman, T. T. Tran, B. D. Ross, P. Bhattacharya, Quality assurance of PASADENA hyperpolarization for ^{13}C biomolecules. *Magn. Reson. Mater. Phy.* **22**, 123–134 (2009).

31. T. Harris, A. Gamliel, S. Uppala, A. Nardi-Schreiber, J. Sosna, J. M. Gomori, R. Katz-Brull, Long-lived ^{15}N hyperpolarization and rapid relaxation as a potential basis for repeated first pass perfusion imaging - Marked effects of deuteration and temperature. *ChemPhysChem* **19**, 2148–2152 (2018).

32. A. Gamliel, D. Shaul, J. M. Gomori, R. Katz-Brull, Signal enhancement of hyperpolarized ^{15}N sites in solution—Increase in solid-state polarization at 3.35 T and prolongation of relaxation in deuterated water mixtures. *NMR Biomed.* **35**, e4787 (2022).

33. D. J. Kushner, A. Baker, T. G. Dunstall, Pharmacological uses and perspectives of heavy water and deuterated compounds. *Can. J. Physiol. Pharmacol.* **77**, 79–88 (1999).

34. R. M. C. Di Martino, B. D. Maxwell, T. Pirali, Deuterium in drug discovery: Progress, opportunities and challenges. *Nat. Rev. Drug Discov.* **22**, 562–584 (2023).

35. J. Eills, R. Picazo-Frutos, D. B. Burueva, L. M. Kovtunova, M. Azagra, I. Marco-Rius, D. Budker, I. V. Koptyug, Combined homogeneous and heterogeneous hydrogenation to yield catalyst-free solutions of parahydrogen-hyperpolarized [1-^{13}C]succinate. *Chem. Commun.* **59**, 9509–9512 (2023).

36. J.-B. Hövener, "^{13}C spin hyperpolarization by PASADENA: Instrumentation, preparation of magnetic tracers, and NMR spectroscopy and imaging in vivo," dissertation, Ruperto-Carola University of Heidelberg, Germany (2008).

37. J.-B. Hövener, E. Y. Chekmenev, K. C. Harris, W. H. Perman, L. W. Robertson, B. D. Ross, P. Bhattacharya, PASADENA hyperpolarization of ^{13}C biomolecules: Equipment design and installation. *Magn. Reson. Mater. Phy.* **22**, 111–121 (2009).

38. E. Chiavazza, E. Kubala, C. V. Gringeri, S. Düwel, M. Durst, R. F. Schulte, M. I. Menzel, Earth's magnetic field enabled scalar coupling relaxation of ^{13}C nuclei bound to fast-relaxing quadrupolar ^{14}N in amide groups. *J. Magn. Reson.* **227**, 35–38 (2013).

39. J. P. Peters, A. Brahms, V. Janicaud, M. Anikeeva, E. Peschke, F. Ellermann, A. Ferrari, D. Hellmold, J. Held-Feindt, N.-M. Kim, J. Meiser, K. Aden, R. Herges, J.-B. Hövener, A. N. Pravdivtsev, Nitrogen-15 dynamic nuclear polarization of nicotinamide derivatives in biocompatible solutions. *Sci. Adv.* **9**, eadd3643 (2023).

40. J. Czernek, R. Fiala, V. Sklenář, Hydrogen bonding effects on the ^{15}N and ^{1}H shielding tensors in nucleic acid base pairs. *J. Magn. Reson.* **145**, 142–146 (2000).

41. S. B. Hakkert, J. Gräfenstein, M. Erdelyi, The ^{15}N NMR chemical shift in the characterization of weak halogen bonding in solution. *Faraday Discuss.* **203**, 333–346 (2017).

42. J. Zhong, J. C. Gore, I. M. Armitage, Relative contributions of chemical exchange and other relaxation mechanisms in protein solutions and tissues. *Magn. Reson. Med.* **11**, 295–308 (1989).

43. R. Kimmich, *NMR: Tomography, Diffusometry, Relaxometry* (Springer-Verlag, 1997).

44. M. Wyszogrodzka, R. Haag, A convergent approach to biocompatible polyglycerol "click" dendrons for the synthesis of modular core-shell architectures and their transport behavior. *Chem. A Eur. J.* **14**, 9202–9214 (2008).

45. M. Dommaschk, F. Gutzeit, S. Boretius, R. Haag, R. Herges, Coordination-induced spin-state-switch (CISSS) in water. *Chem. Commun.* **50**, 12476–12478 (2014).

46. M. Dommaschk, J. Gröbner, V. Wellm, J.-B. Hövener, C. Riedel, R. Herges, Dendronised Ni(II) porphyrins as photoswitchable contrast agents for MRI. *Phys. Chem. Chem. Phys.* **21**, 24296–24299 (2019).

47. W. Jiang, L. Lumata, W. Chen, S. Zhang, Z. Kovacs, A. D. Sherry, C. Khemtong, Hyperpolarized ^{15}N-pyridine derivatives as pH-sensitive MRI agents. *Sci. Rep.* **5**, 9104 (2015).

48. F. Kreis, A. J. Wright, V. Somai, R. Katz-Brull, K. M. Brindle, Increasing the sensitivity of hyperpolarized [$^{15}N_2$]urea detection by serial transfer of polarization to spin-coupled protons. *Magn. Reson. Med.* **84**, 1844–1856 (2020).

49. J. H. Ardenkjær-Larsen, B. Fridlund, A. Gram, G. Hansson, L. Hansson, M. H. Lerche, R. Servin, M. Thaning, K. Golman, Increase in signal-to-noise ratio of >10,000 times in liquid-state NMR. *Proc. Natl. Acad. Sci. U.S.A.* **100**, 10158–10163 (2003).

50. J. Y. C. Lau, L. A. J. Young, T. Zhou, A. Tyler, J. J. J. Miller, D. J. Tyler, "HyperMaple: Maple Syrup as a Glassing Agent for DNP Sample Preparation," *Proc. Intl. Soc. Mag. Reson. Med. 27* (2019), p. 4276. https://cds.ismrm.org/protected/19MProceedings/PDFfiles/4276.html.

51. J. R. Brender, S. Kishimoto, G. R. Eaton, S. S. Eaton, Y. Saida, J. Mitchell, M. C. Krishna, Trehalose as an alternative to glycerol as a glassing agent for in vivo DNP MRI. *Magn. Reson. Med.* **85**, 42–48 (2021).

52. M. Kaushik, H. Lingua, G. Stevanato, M. Elokova, M. Lelli, A. Lesage, O. Ouari, Trehalose matrices for high temperature dynamic nuclear polarization enhanced solid state NMR. *Phys. Chem. Chem. Phys.* **24**, 12167–12175 (2022).

53. I. V. Zhukov, A. S. Kiryutin, A. V. Yurkovskaya, Y. A. Grishin, H.-M. Vieth, K. L. Ivanov, Field-cycling NMR experiments in an ultra-wide magnetic field range: Relaxation and coherent polarization transfer. *Phys. Chem. Chem. Phys.* **20**, 12396–12405 (2018).

54. F. Ellermann, P. Saul, J.-B. Hövener, A. N. Pravdivtsev, Modern manufacturing enables magnetic field cycling experiments and parahydrogen-induced hyperpolarization with a benchtop NMR. *Anal. Chem.* **95**, 6244–6252 (2023).

55. J. P. Peters, C. D. Assaf, J.-B. Hövener, A. N. Pravdivtsev, Compact magnetic field cycling in the range from nT to 9.4 T exemplified with ^{13}C relaxation dispersion and SABRE-SHEATH hyperpolarization. arXiv:2506.08711 [physics.chem-ph] (2025).

56. A. S. Kiryutin, A. N. Pravdivtsev, K. L. Ivanov, Y. A. Grishin, H.-M. Vieth, A. V. Yurkovskaya, A fast field-cycling device for high-resolution NMR: Design and application to spin relaxation and hyperpolarization experiments. *J. Magn. Reson.* **263**, 79–91 (2016).

57. P. Miéville, S. Jannin, G. Bodenhausen, Relaxometry of insensitive nuclei: Optimizing dissolution dynamic nuclear polarization. *J. Magn. Reson.* **210**, 137–140 (2011).

58. N. Chattergoon, F. Martínez-Santiesteban, W. B. Handler, J. H. Ardenkjær-Larsen, T. J. Scholl, Field dependence of T_1 for hyperpolarized [1-^{13}C]pyruvate. *Contrast Media Mol. Imaging* **8**, 57–62 (2013).

59. L. Hwang, J. H. Freed, Dynamic effects of pair correlation functions on spin relaxation by translational diffusion in liquids. *J. Chem. Phys.* **63**, 4017–4025 (1975).

60. C. F. Polnaszek, R. G. Bryant, Nitroxide radical induced solvent proton relaxation: Measurement of localized translational diffusion. *J. Chem. Phys.* **81**, 4038–4045 (1984).

61. M. R. Popović-Nikolić, G. V. Popović, K. Stojilković, M. Dobrosavljević, D. D. Agbaba, Acid–base equilibria of rupatadine fumarate in aqueous media. *J. Chem. Eng. Data* **63**, 3150–3156 (2018).

62. J. C. Facelli, Nitrogen-15 chemical shift in the pyridine–methanol complex. *Chem. Phys. Lett.* **322**, 91–96 (2000).

63. K. Mizuno, Y. Miyashita, Y. Shindo, H. Ogawa, NMR and FT-IR studies of hydrogen bonds in ethanol-water mixtures. *J. Phys. Chem.* **99**, 3225–3228 (1995).

64. Z. Dega-Szafran, M. Szafran, J. Sitkowski, L. Stefaniak, Nitrogen-15 NMR studies of hydrogen bonding and proton transfer in complexes of pyridine N-oxides with dichloroacetic acid in $CDCl_3$. *J. Phys. Orga. Chem.* **9**, 746–750 (1996).

65. P. Pham, C. Hilty, R_2 relaxometry of SABRE-hyperpolarized substrates at a low magnetic field. *Anal. Chem.* **95**, 16911–16917 (2023).

66. C. Qi, O. Mankinen, V.-V. Telkki, C. Hilty, Measuring protein-ligand binding by hyperpolarized ultrafast NMR. *J. Am. Chem. Soc.* **146**, 5063–5066 (2024).

67. J. Kowalewski, L. Mäler, *Nuclear Spin Relaxation in Liquids: Theory, Experiments, and Applications* (Taylor & Francis, 2006).

68. N.-S. Cheng, Formula for the viscosity of a glycerol−water mixture. *Ind. Eng. Chem. Res.* **47**, 3285–3288 (2008).

69. K. Takamura, H. Fischer, N. R. Morrow, Physical properties of aqueous glycerol solutions. *J. Petrol. Sci. Eng.* **98-99**, 50–60 (2012).

70. G. Amudha, R. Santhakumari, D. Chandrika, S. Mugeshini, N. Rajeswari, S. Sagadevan, Growth, spectroscopic and Hirshfeld surface analysis on pyridine urea single crystal. *J. Mol. Struct.* **1257**, 132606 (2022).

71. L. Plasseraud, Glycerol as ligand in metal complexes—A structural review. *Crystals* **14**, 217 (2024).

72. M. H. Abraham, P. P. Duce, D. V. Prior, D. G. Barratt, J. J. Morris, P. J. Taylor, Hydrogen bonding. Part 9. Solute proton donor and proton acceptor scales for use in drug design. *J. Chem. Soc., Perkin Trans. 2*, 1355–1375 (1989).

73. P. M. Schaber, J. Colson, S. Higgins, D. Thielen, B. Anspach, J. Brauer, Thermal decomposition (pyrolysis) of urea in an open reaction vessel. *Thermochim. Acta* **424**, 131–142 (2004).

74. D. O. Guarin, S. M. Joshi, A. Samoilenko, M. S. H. Kabir, E. E. Hardy, A. M. Takahashi, J. H. Ardenkjaer-Larsen, E. Y. Chekmenev, Y.-F. Yen, Development of dissolution dynamic nuclear polarization of [15N3]metronidazole: A clinical approved antibiotic. *Angew. Chem. Int. Ed. Engl.* **135**, e202219181 (2023).

75. S. Nikolaus, B. Schulte, N. Al-Massad, F. Thieme, D. M. Schulte, J. Bethge, A. Rehman, F. Tran, K. Aden, R. Häsler, N. Moll, G. Schütze, M. J. Schwarz, G. H. Waetzig, P. Rosenstiel, M. Krawczak, S. Szymczak, S. Schreiber, Increased tryptophan metabolism is associated with activity of inflammatory bowel diseases. *Gastroenterology* **153**, 1504–1516.e2 (2017).

76. K. Kouřil, M. Gramberg, M. Jurkutat, H. Kouřilová, B. Meier, A cryogen-free, semi-automated apparatus for bullet-dynamic nuclear polarization with improved resolution. *Magn. Reson.* **2**, 815–825 (2021).

77. M. Ceillier, O. Cala, T. E. Daraï, S. F. Cousin, Q. Stern, S. Guibert, S. J. Elliott, A. Bornet, B. Vuichoud, J. Milani, C. Pages, D. Eshchenko, J. G. Kempf, C. Jose, S. A. Lambert, S. Jannin, An automated system for fast transfer and injection of hyperpolarized solutions. *J. Magn. Reson. Open* **8–9**, 100017 (2021).

78. H. Shang, T. Skloss, C. von Morze, L. Carvajal, M. Van Criekinge, E. Milshteyn, P. E. Z. Larson, R. E. Hurd, D. B. Vigneron, Handheld electromagnet carrier for transfer of hyperpolarized carbon-13 samples. *Magn. Reson. Med.* **75**, 917–922 (2016).

79. A. Z. Lau, J. J. Miller, M. D. Robson, D. J. Tyler, Cardiac perfusion imaging using hyperpolarized 13c urea using flow sensitizing gradients. *Magn. Reson. Med.* **75**, 1474–1483 (2016).

80. R. V. Shchepin, D. A. Barskiy, D. M. Mikhaylov, E. Y. Chekmenev, Efficient synthesis of nicotinamide-1-^{15}N for ultrafast NMR hyperpolarization using parahydrogen. *Bioconjug. Chem.* **27**, 878–882 (2016).

81. G. R. Fulmer, A. J. M. Miller, N. H. Sherden, H. E. Gottlieb, A. Nudelman, B. M. Stoltz, J. E. Bercaw, K. I. Goldberg, NMR chemical shifts of trace impurities: Common laboratory

solvents, organics, and gases in deuterated solvents relevant to the organometallic chemist. *Organometallics* **29**, 2176–2179 (2010).
